# Pembrolizumab for advanced urothelial carcinoma: exploratory ctDNA biomarker analyses of the KEYNOTE-361 phase 3 trial

**DOI:** 10.1038/s41591-024-03091-7

**Published:** 2024-06-01

**Authors:** Thomas Powles, Yen-Hwa Chang, Yoshiaki Yamamoto, Jose Munoz, Felipe Reyes-Cosmelli, Avivit Peer, Graham Cohen, Evan Y. Yu, Anja Lorch, Abhishek Bavle, Blanca Homet Moreno, Julia Markensohn, Mackenzie Edmondson, Cai Chen, Razvan Cristescu, Carol Peña, Jared Lunceford, Seyda Gunduz

**Affiliations:** 1 Barts Cancer Institute, Queen Mary University of London, London, UK; 2 Taipei Veterans General Hospital, Taipei, Taiwan; 3 Yamaguchi University Hospital, Yamaguchi, Japan; 4 Hospital Universitari i Politècnic La Fe, Valencia, Spain; 5 Fundación Arturo López Pérez, Santiago, Chile; 6 Rambam Health Care Campus, Haifa, Israel; 7 Mary Potter Oncology Centre, Gauteng, South Africa; 8 Fred Hutchinson Cancer Center and University of Washington, Seattle, WA USA; 9 Universitätsspital Zürich, Zürich, Switzerland; 10 University Hospital Düsseldorf, Düsseldorf, Germany; 11 Merck & Co. Inc., Rahway, NJ USA; 12 Istinye University Liv Hospital, Istanbul, Turkey

**Keywords:** Prognostic markers, Bladder cancer

## Abstract

Circulating tumor DNA (ctDNA) is emerging as a potential biomarker in early-stage urothelial cancer, but its utility in metastatic disease remains unknown. In the phase 3 KEYNOTE-361 study, pembrolizumab with and without chemotherapy was compared with chemotherapy alone in patients with metastatic urothelial cancer. The study did not meet prespecified efficacy thresholds for statistical significance. To identify potential biomarkers of response, we retrospectively evaluated the association of pre- and posttreatment ctDNA with clinical outcomes in a subset of patients who received pembrolizumab (*n* = 130) or chemotherapy (*n* = 130) in KEYNOTE-361. Baseline ctDNA was associated with best overall response (BOR; *P* = 0.009), progression-free survival (*P* < 0.001) and overall survival (OS; *P* < 0.001) for pembrolizumab but not for chemotherapy (all; *P* > 0.05). Chemotherapy induced larger ctDNA decreases from baseline to treatment cycle 2 than pembrolizumab; however, change with pembrolizumab (*n* = 87) was more associated with BOR (*P* = 4.39 × 10^−5^) and OS (*P* = 7.07 × 10^−5^) than chemotherapy (*n* = 102; BOR: *P* = 1.01 × 10^−4^; OS: *P* = 0.018). Tumor tissue-informed versions of ctDNA change metrics were most associated with clinical outcomes but did not show a statistically significant independent value for explaining OS beyond radiographic change by RECIST v.1.1 when jointly modeled (pembrolizumab *P* = 0.364; chemotherapy *P* = 0.823). These results suggest distinct patterns in early ctDNA changes with immunotherapy and chemotherapy and differences in their association with long-term outcomes, which provide preliminary insights into the utility of liquid biopsies for treatment monitoring in metastatic urothelial cancer. Clinical trial registration: NCT02853305.

## Abstract (web summary)

In an exploratory analysis of the KEYNOTE-361 phase 3 trial that tested immunotherapy and chemotherapy in advanced urothelial carcinoma, different early circulating tumor DNA (ctDNA) dynamics were observed between the two treatments, and ctDNA levels were associated with response to pembrolizumab but not to chemotherapy.

## Main

Circulating tumor DNA (ctDNA) is cell-free DNA that enters the bloodstream after being released by tumor cells during apoptosis, necrosis and other mechanisms of cell death^1^. ctDNA has a short half-life and can be detected noninvasively through liquid biopsies, making it a useful marker of disease^2^. The utility of ctDNA has been studied extensively in the last decade and may have a role in many applications of clinical management, including cancer screening, prognosis, early recurrence detection, estimation of tumor burden, treatment decisions and monitoring for treatment benefit^3–5^. Assessment of ctDNA can also determine tumor-specific mutations and thus aid in patient selection for treatment^6^. Several ctDNA-based assays are available for mutation detection for treatment selection (including, but not limited to, *EGFR*, *BRCA1/2* and *KRAS*^*G12C*^ mutations), but currently no ctDNA-based minimal residual disease test has been approved or cleared by the US Food and Drug Administration (FDA). Signatera (Natera, Inc.) and, more recently, other ctDNA-based minimal residual disease tests have received Medicare reimbursement for monitoring disease and response to treatment in colorectal, breast and ovarian cancers as well as muscle-invasive bladder cancer (MIBC), although the focus of these applications has been mostly in early-stage disease^7^.

ctDNA has been shown to have a role in reflecting response to treatment in solid tumors as well as potential prognostic implications, although varying results have been reported in the treatment of different tumor types and degrees of tumor burden^8,9^. In pancreatic cancer, high pretreatment ctDNA levels correlated with higher tumor burden and poorer survival^10^. By contrast, a study in advanced anal squamous cell carcinoma reported no prognostic impact of ctDNA detection at baseline^11^. Regarding the monitoring of early ctDNA dynamics, ctDNA changes have been associated with responses to first-line therapy in patients with advanced non-small cell lung cancer^12^. A significant correlation was observed between the ctDNA percentage change at the first follow-up and the percentage change in tumor target lesion size from baseline (*R* = 0.66; *P* < 0.001)^12^. Other studies have demonstrated that ctDNA detection may be influenced by the stage of disease. One study reported detectable ctDNA in up to 75% of patients with advanced solid tumors compared with a range of 48–73% in localized tumors^13^. In patients with non-small cell lung cancer, pretreatment ctDNA was detected in 42%, 67% and 88% of patients with stage I, II and III disease, respectively^14^.

For bladder cancer, the relationship between ctDNA and clinical outcomes has been demonstrated mostly in early-stage disease. One study evaluated the prognostic value of ctDNA in predicting recurrence in patients with MIBC who achieved a pathological complete response (pCR) after neoadjuvant chemotherapy before cystectomy and reported that the absence of ctDNA at baseline was associated significantly with pCR (*P* < 0.0001)^15^. Furthermore, ctDNA status at baseline and before cystectomy was a better predictor of recurrence-free survival compared with pCR (both *P* < 0.0001)^15,16^. In a systematic review of MIBC, ctDNA was suggested to be a prognostic factor following radical cystectomy and may be used to monitor recurrence^17^. A prospective study of MIBC evaluating the utility of ctDNA to detect metastatic relapse after cystectomy and treatment efficacy reported significantly higher ctDNA levels in patients with metastatic relapse compared with patients who were disease free (*P* < 0.001)^18^.

Clinical outcomes in urothelial carcinoma have improved with the availability of immunotherapies, but survival rates remain poor^19^; thus, the identification of biomarkers to aid in treatment decisions is of interest. In the IMVigor010 study, a strong prognostic significance of postsurgery ctDNA status was demonstrated for high-risk MIBC; patients who were positive for ctDNA at study enrollment in either the atezolizumab or observation arm had significantly worse outcomes (shorter disease-free survival and OS) than those who were ctDNA negative following surgery^20^. In the ctDNA-positive group, improvements in disease-free survival (hazard ratio (HR), 0.58 (95% confidence interval (CI), 0.43–0.79)) and OS (HR, 0.59; 95% CI, 0.41–0.86) were found with atezolizumab versus observation^20^. In urothelial carcinoma, ctDNA could be particularly useful in the context of evaluating the responses of patients being treatment with targeted therapy. In the phase 1b BISCAY trial, which enrolled patients with advanced urothelial carcinoma, a ctDNA analysis was conducted to evaluate genomic alterations in patients treated with durvalumab and select targeted therapies using an informed-based panel analysis (GuardantOMNI)^21^. In patients who received durvalumab plus fibroblast growth factor receptor (FGFR) inhibitors, a correlation was found between ctDNA and tissue for *FGFR* DNA alterations, and changes to *FGFR* mutations were associated with clinical outcomes^21^. Analysis of ctDNA in advanced or metastatic urothelial carcinoma has been used largely in genomic profiling^22^, and its utility as a prognostic factor or marker for monitoring disease in metastatic urothelial carcinoma remains unknown.

KEYNOTE-361 is a randomized, open-label, phase 3 trial that evaluated first-line pembrolizumab with or without platinum-based chemotherapy compared with platinum-based chemotherapy in patients with advanced urothelial carcinoma^23^. The prespecified threshold for statistical significance for pembrolizumab plus chemotherapy versus chemotherapy alone for either primary endpoint was not met (progression-free survival (PFS); HR, 0.78, 95% CI 0.65–0.93; *P* = 0.0033; OS: HR, 0.86; 95% CI, 0.72–1.02; *P* = 0.0407)^23^. In an exploratory analysis of KEYNOTE-361, tissue tumor mutational burden (tTMB) was positively associated with clinical outcomes with pembrolizumab monotherapy^24^. Determining the utility of ctDNA as a biomarker in patients with advanced or metastatic urothelial carcinoma from KEYNOTE-361 is of interest.

We designed a prespecified retrospective exploratory analysis of ctDNA in a subset of patients from the pembrolizumab monotherapy arm and the chemotherapy alone arm of KEYNOTE-361 to determine the association between ctDNA and clinical outcomes. ctDNA was assessed using the Guardant OMNI ctDNA panel, which uses a proprietary molecular response score to report ctDNA changes. Three additional metrics for quantitative evaluation of ctDNA were also considered. We were interested in assessing the performance of a tumor-independent ctDNA assay in the metastatic setting to determine whether a tumor-independent approach would have meaningful relationships with clinical outcomes. To optimize the relationship between a panel-based ctDNA test and clinical outcomes, we used several different metrics to calculate ctDNA levels, including maximum variant allele frequency (VAF) (maxVAF), mean VAF (meanVAF) and a proprietary molecular response (MR) score provided by Guardant Health (GH) with their GuardantOMNI assay output. In addition, we explored whether the relationship with clinical outcomes observed using the tumor-independent metrics would be similar to a tumor-informed approach, which was simulated in the present study by using already available tumor tissue and normal whole-exome sequencing (WES) data from these patients to select a subset of the panel-identified variants (those identified in the tissue WES) to define ctDNA levels. The overarching aims were to determine whether a set of baseline and change-from-baseline ctDNA metrics were associated with clinical outcomes and to evaluate the patterns of ctDNA response under treatment with immunotherapy (that is, pembrolizumab) versus chemotherapy. A statistical analysis plan was predefined and followed for this analysis.

## Results

**Extended Data Fig. 1:**
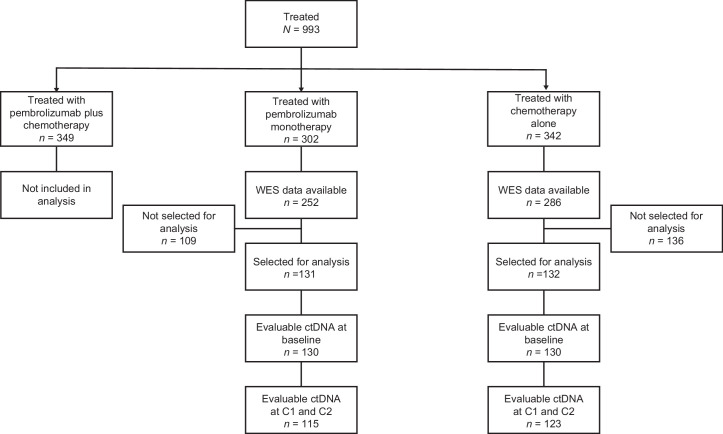
Trial profile. C1, cycle 1; C2, cycle 2; ctDNA, circulating tumor DNA; WES, whole-exome sequencing.

**Extended Data Fig. 2:**
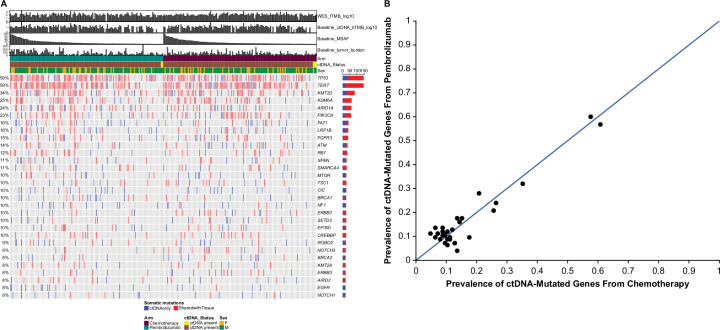
OncoPrint illustration of baseline ctDNA. (**a**) OncoPrint illustration in the chemotherapy and pembrolizumabs arm and (**b**) prevalence in the chemotherapy arm versus pembrolizumab arm for the top 30 mutated genes (each dot below is one gene) from baseline ctDNA data.

Between 19 October 2016 and 29 June 2018, 1,010 patients were randomly assigned to receive pembrolizumab plus chemotherapy, pembrolizumab monotherapy or chemotherapy. Median follow-up, defined as time from randomization to data cutoff date (29 April 2020), in the intention-to-treat population was 31.7 months (interquartile range, 27.7–36.0). Of the patients who received one or more doses of treatment, 302 received pembrolizumab monotherapy and 342 received chemotherapy. Of these patients, 538 had WES data available, and 263 were selected for this analysis in a manner achieving a representative subset in terms of clinical outcomes, baseline demographics and key biomarkers (tTMB and PD-L1 combined positive score (CPS)). Of the selected patients, 260 had evaluable ctDNA at baseline (pembrolizumab, *n* = 130; chemotherapy, *n* = 130) and 238 had ctDNA data evaluable at both baseline and treatment cycle 2 (C2) (pembrolizumab, *n* = 115; chemotherapy, *n* = 123; Extended Data Fig. 1). Clinical characteristics and baseline ctDNA levels within arms were generally similar (Extended Data Table 1). The ctDNA mutational landscape showed no obvious differences between the two arms (Extended Data Fig. 2).

### Baseline

**Fig. 1:**
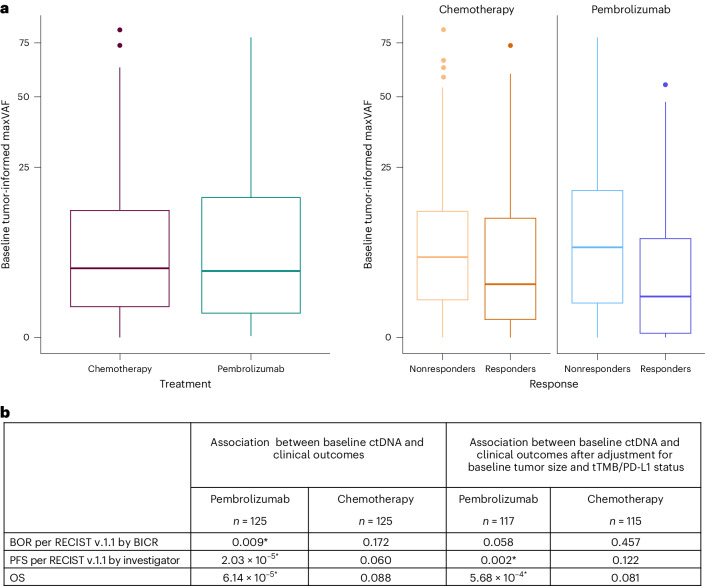
Baseline ctDNA assessment by tumor-informed maxVAF and association with clinical outcomes. **a**, Patient-level baseline tumor-informed maxVAF by response status and treatment arm (pembrolizumab, *n* = 125; chemotherapy, *n* = 125). The center line corresponds to the median, and the box is delineated by first and third quartiles. Whiskers extend to any points within 1.5 times the interquartile range, with points lying beyond identified individually as potential outliers. **b**, Association between baseline ctDNA and clinical outcomes by tumor-informed maxVAF evaluated using logistic regression (BOR) and Cox proportional hazards regression (PFS and OS), with adjustment for ECOG PS. Multiplicity-adjusted *P* values were calculated. Significance was prespecified at *α* = 0.05. Asterisks indicate significance. Hypothesis: negative association.

**Extended Data Fig. 3:**
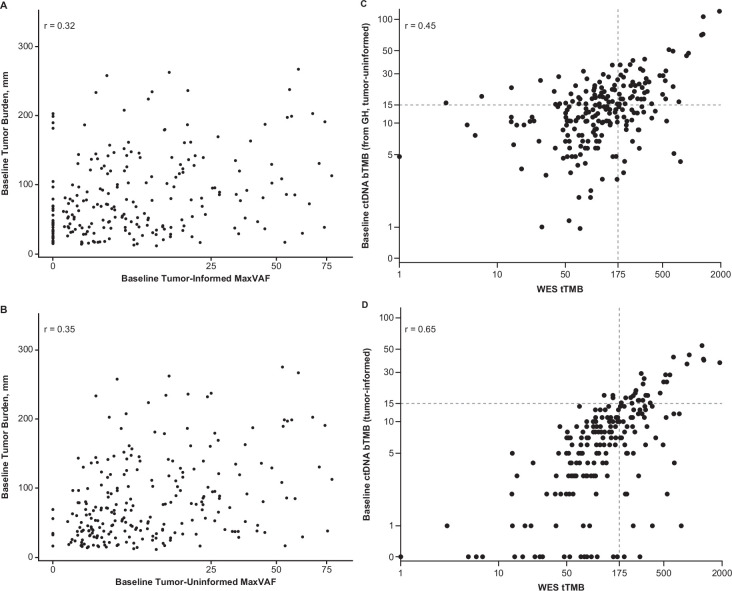
Correlation between tumor burden and tumor-informed maxVAF and between bTMB and WES tTMB. (**a**) Correlation between baseline tumor burden and baseline tumor-informed (N = 232) maxVAF; (**b**) correlation between baseline tumor burden and baseline tumor-uninformed (N = 240) maxVAF; (**c**) Correlation between tumor-uninformed bTMB versus WES tTMB (N = 218); (**d**) correlation between baseline ctDNA tumor informed bTMB versus WES tTMB (N = 218). maxVAF, maximum variant allele frequency; tTMB, tissue tumor mutational burden; WES, whole-exome sequencing.

At baseline, 80.8% of patients in the pembrolizumab arm and 87.2% of patients in the chemotherapy arm were ctDNA-positive per tumor-informed maxVAF (Extended Data Table 1). A moderate positive correlation was observed between baseline tumor burden and baseline tumor-informed and tumor-uninformed maxVAF (Extended Data Fig. 3). A weak correlation (Spearman correlation coefficient, *r* = 0.45) was observed between baseline tumor-uninformed blood-derived TMB (bTMB) and WES tTMB, with an improved correlation using the tumor-informed ctDNA dataset (Spearman correlation coefficient, *r* = 0.65) (Extended Data Fig. 3). In the pembrolizumab arm, lower baseline tumor-informed maxVAF was associated with improved BOR (*P* = 0.009), PFS (*P* < 0.001) and OS (*P* < 0.001; Fig. 1). In the chemotherapy arm, lower baseline tumor-informed maxVAF was not associated with improved BOR, PFS or OS (all, *P* > 0.05), although a trend in the hypothesized direction was observed. In the pembrolizumab arm, the association of baseline tumor-informed maxVAF with PFS and OS were robust to the adjustment for baseline tumor size and tTMB and PD-L1 status in both metrics (*P* < 0.01); BOR was no longer associated after adjustment (*P* > 0.05; Fig. 1). In the chemotherapy arm, lower baseline tumor-informed maxVAF remained unassociated with clinical outcomes (all; *P* > 0.05) after adjustment for baseline tumor size and tTMB and PD-L1 status. Results were similar for tumor-uninformed maxVAF (Supplementary Fig. 1).

**Extended Data Fig. 4:**
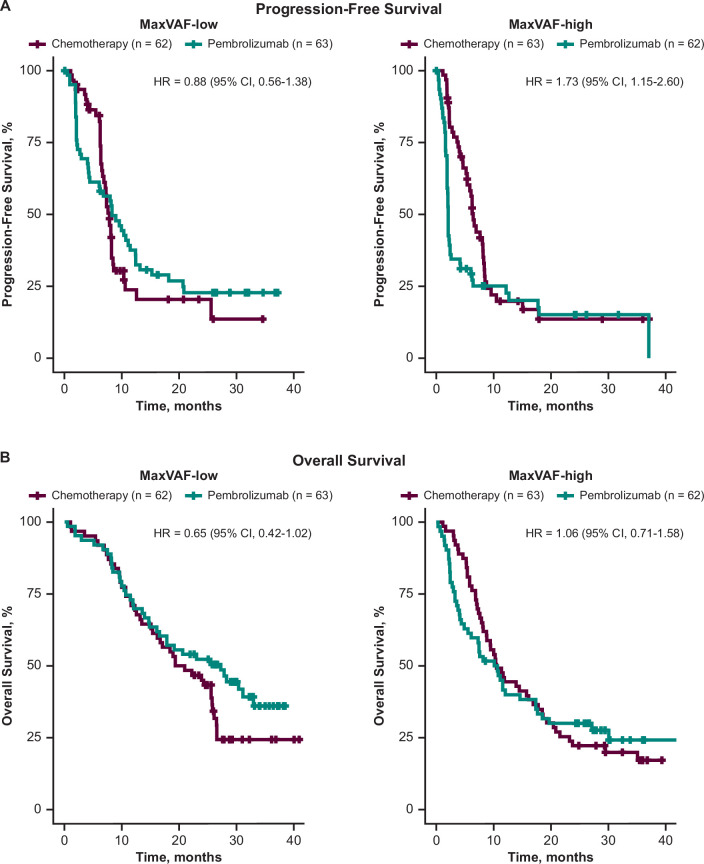
Survival by low versus high baseline tumor-informed maxVAF, Kaplan-Meier estimates. (**a**) PFS and (**b**) OS. maxVAF, maximum variant allele frequency; OS, overall survival; PFS, progression-free survival. Median cutoff 3.84.

**Extended Data Fig. 5:**
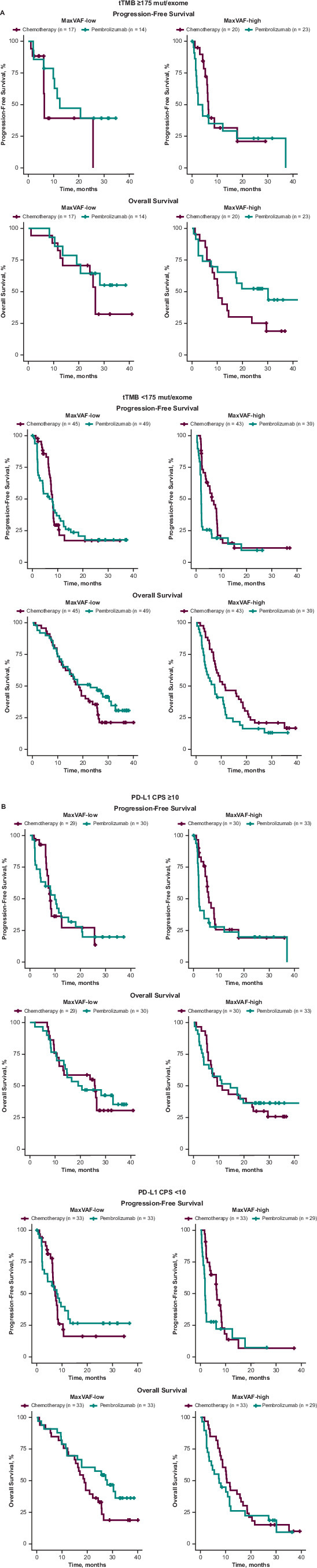
Survival by low and high baseline tumor-informed maxVAF and tTMB and PD-L1 status. Kaplan-Meier estimates of PFS and OS by low versus high baseline tumor-informed maxVAF by (a) tTMB and (b) PD-L1 status. (**a**) tTMB and (**b**) PD-L1 status.maxVAF, maximum variant allele frequency; OS, overall survival; PD-L1, programmed cell death ligand 1; PFS, progression-free survival; tTMB, tissue tumor mutational burden. Median cutoff 3.84.

Using the median baseline tumor-informed maxVAF for illustrative subgroup analyses, more favorable trends for pembrolizumab versus chemotherapy were observed with HR estimates in patients with ‘low’ baseline tumor-informed maxVAF (Extended Data Fig. 4). However, trends observed in baseline tumor-informed maxVAF subgroups were also influenced by TMB and PD-L1 status (Extended Data Fig. 5).

### Monitoring

**Fig. 2:**
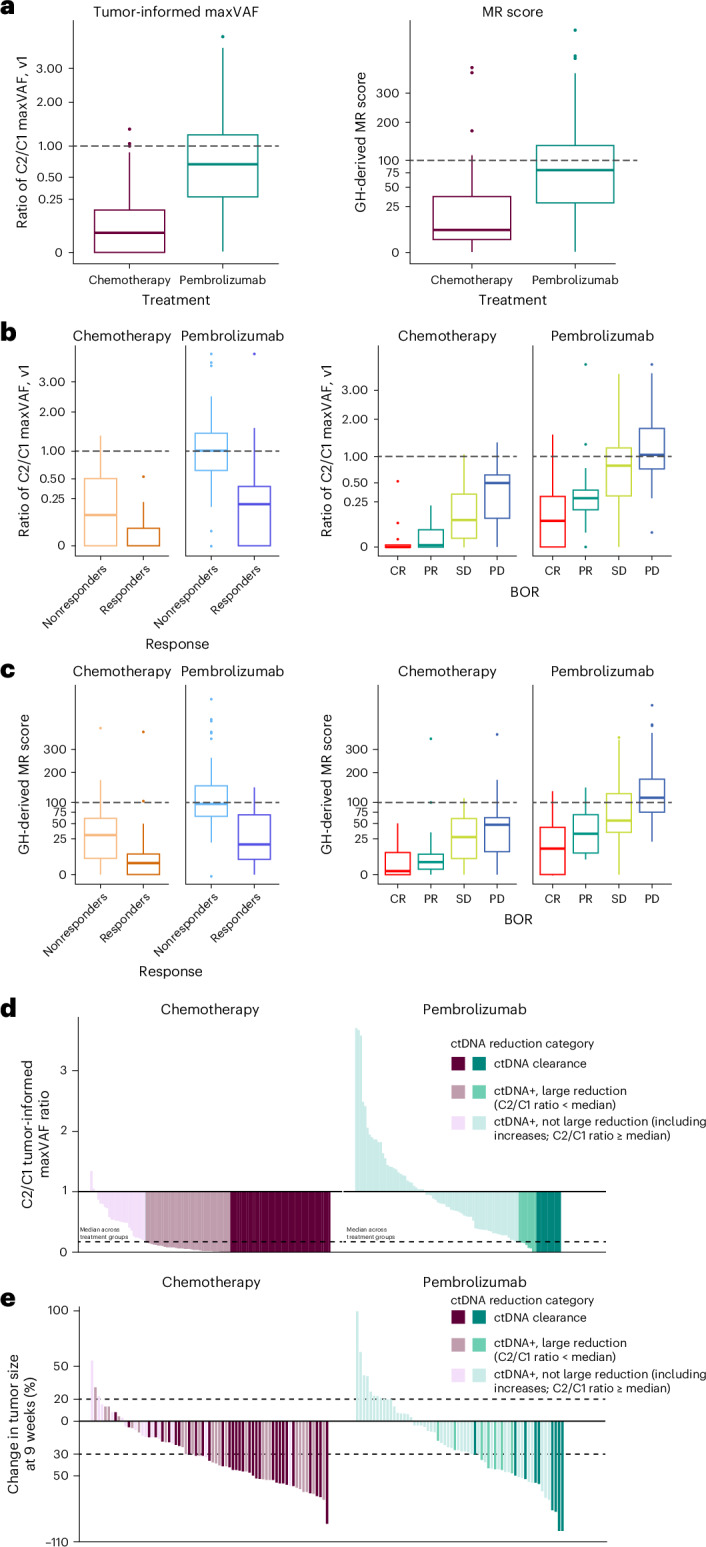
C2/C1 ctDNA assessment for tumor-informed maxVAF and MR scores. **a**, Overall C2/C1 tumor-informed maxVAF (pembrolizumab, *n* = 87; chemotherapy, *n* = 102) and MR score changes (pembrolizumab, *n* = 89; chemotherapy, *n* = 89) by treatment arm. **b**, Patient-level C2/C1 tumor-informed maxVAF changes (pembrolizumab, *n* = 87; chemotherapy, *n* = 102) by response status, BOR and treatment arm. **c**, MR score changes (pembrolizumab, *n* = 89; chemotherapy, *n* = 89) by response status, BOR and treatment arm. **d**,**e**, C2/C1 tumor-informed maxVAF ratio (**d**) and best 9-week percentage change (**e**) from baseline in tumor size for the pembrolizumab and the chemotherapy arms. In **a**–**c**, the center line corresponds to the median and the box is delineated by first and third quartiles. Whiskers extend to any points within 1.5 times the interquartile range, with points lying beyond identified individually as potential outliers. CR, complete response; PD, progressive disease; PR, partial response; SD, stable disease.

At C2, 11.5% of patients in the pembrolizumab arm and 41.2% of patients in the chemotherapy arm had ctDNA clearance by tumor-informed maxVAF (Extended Data Table 2). Smaller C2 reductions in ctDNA levels relative to C1 were observed in the pembrolizumab versus chemotherapy arm (median ratio of C2/C1 tumor-informed maxVAF, 0.71 versus 0.03, respectively; Fig. 2a,d). Similar findings were observed for radiographic tumor shrinkage in this patient population (Fig. 2e). Greater separation was observed in the distribution of tumor-informed maxVAF changes for responders than for nonresponders in the pembrolizumab arm and nonresponders had no shift from baseline (Fig. 2b). In the chemotherapy arm, nonresponders still had substantial decreases in tumor-informed maxVAF at C2. Larger C2/C1 maxVAF decreases for each RECIST (Response Evaluation Criteria in Solid Tumors) v.1.1 response category were observed for the chemotherapy arm relative to the pembrolizumab arm (Fig. 2b). Results were similar with MR score, tumor-uniformed maxVAF and tumor-uninformed meanVAF changes (Fig. 2c and Supplementary Fig. 2). Changes from baseline were associated with clinical outcome (BOR, PFS and OS) in both arms, although results were not always consistent across different methodologies (tumor-informed maxVAF/MR). The association between ctDNA change and outcome was stronger for pembrolizumab, especially for OS, which is the most robust clinical endpoint in advanced urothelial carcinoma^25^ (Table 1; Supplementary Table 1). Associations were robust to adjustment for tTMB and PD-L1 status (Table 1). Of the VAF metrics evaluated, only the tumor-informed version showed significant associations for all three clinical outcomes (BOR, PFS and OS) in the chemotherapy arm (Table 1 and Supplementary Table 1). In the chemotherapy arm, tumor-informed maxVAF reductions were associated with improved BOR (*P* < 0.001), PFS (*P* < 0.001) and OS (*P* < 0.05; Table 1). MR score was associated with improved BOR and PFS (*P* < 0.01) but not OS (*P* > 0.05). Results were similar for C2/C1 tumor-uninformed maxVAF and tumor-uninformed meanVAF changes (Supplementary Table 1).

**Table 1:** Association between C2/C1 ctDNA change and clinical outcomes by tumor-informed maxVAF and MR score

	Tumor-informed maxVAF				MR score			
Outcomes	Association between C2/C1 ctDNA change and clinical outcomes		Association between C2/C1 ctDNA change and clinical outcomes after adjustment for tTMB and PD-L1 status		Association between C2/C1 ctDNA change and clinical outcomes		Association between C2/C1 ctDNA change and clinical outcomes after adjustment for tTMB and PD-L1 status	
	Pembrolizumab	Chemotherapy	Pembrolizumab	Chemotherapy	Pembrolizumab	Chemotherapy	Pembrolizumab	Chemotherapy
	*n* = 87	*n* = 102	*n* = 87	*n* = 102	*n* = 89	*n* = 89	*n* = 89	*n* = 89
BOR per RECIST v.1.1 by BICR	4.39 × 10^−5^*	1.01 × 10^−4^*	7.83 × 10^−5^*	1.34 × 10^−4^*	5.85 × 10^−4^*	0.001*	1.04 × 10^−4^*	0.002*
PFS per RECIST v.1.1 by investigator	1.30 × 10^−6^*	1.10 × 10^−6^*	1.61 × 10^−5^*	1.20 × 10^−6^	3.60 × 10^−6^*	0.007*	3.49 × 10^−5^*	0.008*
OS	7.07 × 10^−5*^	0.018*	0.002*	0.011*	7.07 × 10^−5^*	0.264	0.001*	0.260

Association between C2/C1 ctDNA change and clinical outcomes after adjustment for tTMB and PD-L1 status and BOR				
	Tumor-informed maxVAF		MR score	
Outcomes	Pembrolizumab	Chemotherapy	Pembrolizumab	Chemotherapy
	*n* = 87	*n* = 102	*n* = 89	*n* = 89
PFS per RECIST v.1.1 by investigator	0.046*	8.65 × 10^−4^*	0.129	0.526
OS	0.364	0.823	0.364	0.823

Association was evaluated using logistic regression (BOR) and Cox proportional hazards regression (PFS and OS), with adjustment for ECOG PS. Multiplicity-adjusted *P* values were calculated. Significance was prespecified at *α* = 0.05. Asterisks indicate significance. Hypothesis: one-sided alternative hypothesis testing for a negative association.

**Fig. 3:**
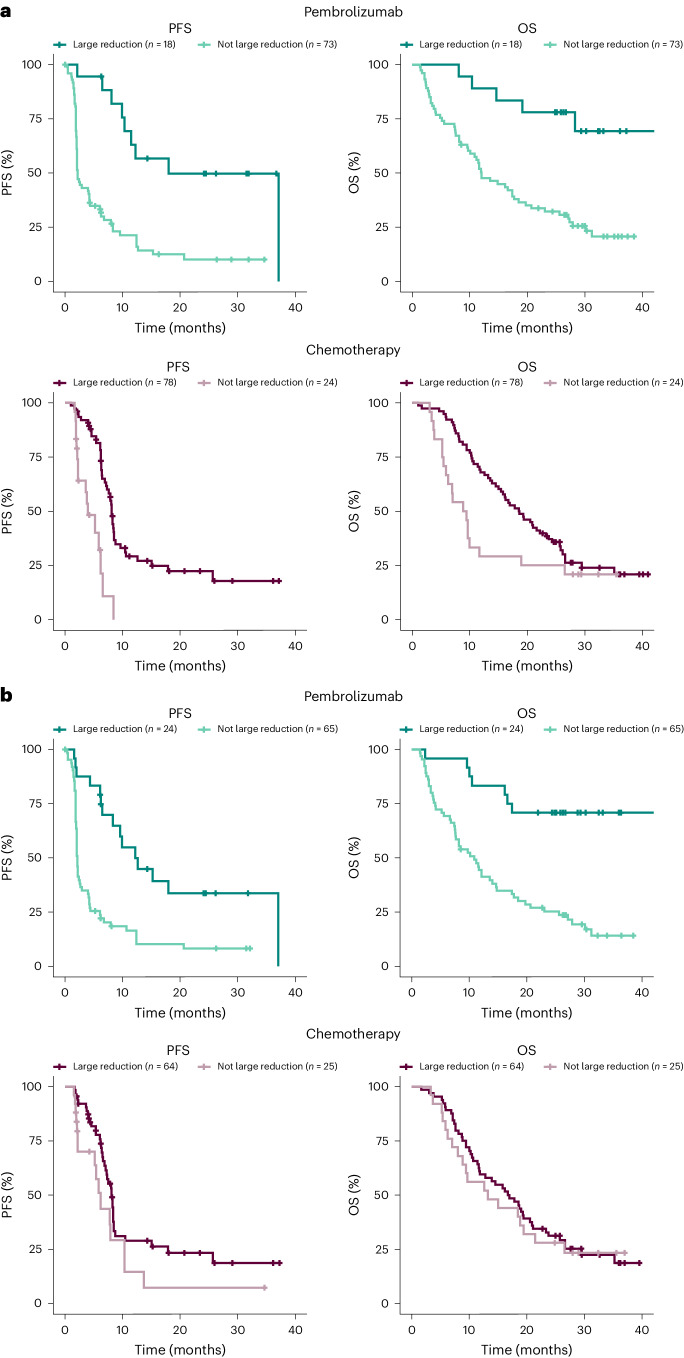
Survival by large and not large C2/C1 reductions within the pembrolizumab and chemotherapy arms. **a**,**b**, Kaplan–Meier estimates of survival by C2/C1 tumor-informed maxVAF (**a**) and MR score changes (**b**). Large reduction = C2/C1 ratio below the median; not large = C2/C1 ratio above the median. Median cutoff 0.18 (**a**), 34 (**b**).

**Extended Data Fig. 6:**
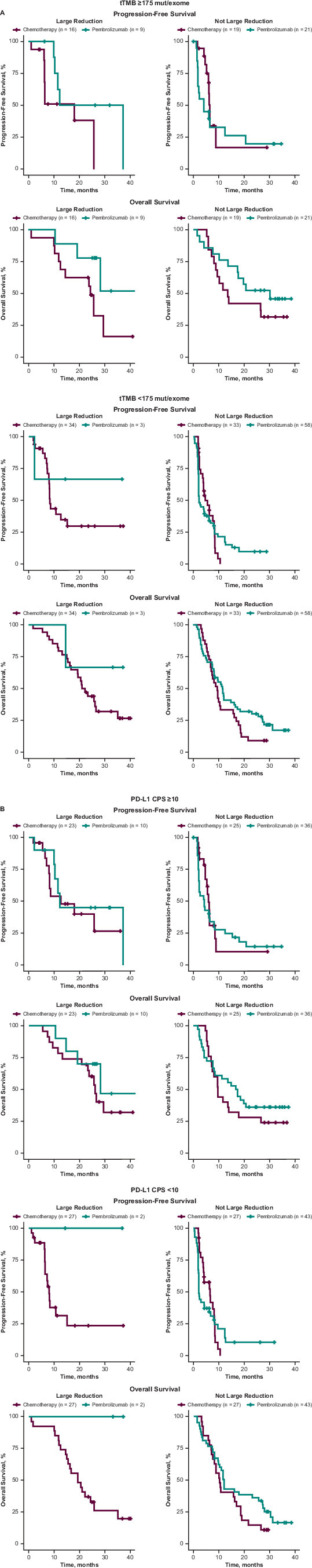
Survival by large versus not large C2/21 tumor-informed maxVAF and tTMB and PD-L1 status. Kaplan-Meier estimates of PFS and OS by C2/C1 by (a) tTMB and (b) PD-L1 status. C1, cycle 1; C2, cycle 2; CPS, combined positive score; maxVAF, maximum variant allele frequency; OS, overall survival; PD-L1, programmed cell death ligand 1; PFS, progression-free survival; tTMB, tissue tumor mutational burden. Median cutoff 0.18; Large reduction = C2/C1 ratio below the median; Not Large = C2/C1 ratio above the median.

Kaplan–Meier survival estimates using the tumor-informed maxVAF and MR score illustrated the differences in PFS and OS within treatment arm using the median ctDNA reduction as an illustrative cutoff. Results were consistent with the hypothesis testing conclusions, with more dramatic separation observed in the pembrolizumab arm than in the chemotherapy arm (Fig. 3). Results may also be impacted by tTMB ≥175 mutations per exome (mut/exome) and PD-L1 CPS ≥ 10 status (Extended Data Fig. 6). When RECIST v.1.1 response status was added as a variable, ctDNA changes from C1 to C2 were no longer significantly associated with OS in either arm (*P* > 0.05; Table 1 and Supplementary Table 1). Adjusting for 9-week percentage change in tumor size led to a similar finding (Extended Data Table 3). Response by RECIST v.1.1 and 9-week percentage change in tumor size retained statistical significance (nominal *P* < 0.05) in such joint models (Extended Data Table 4).

**Fig. 4:**
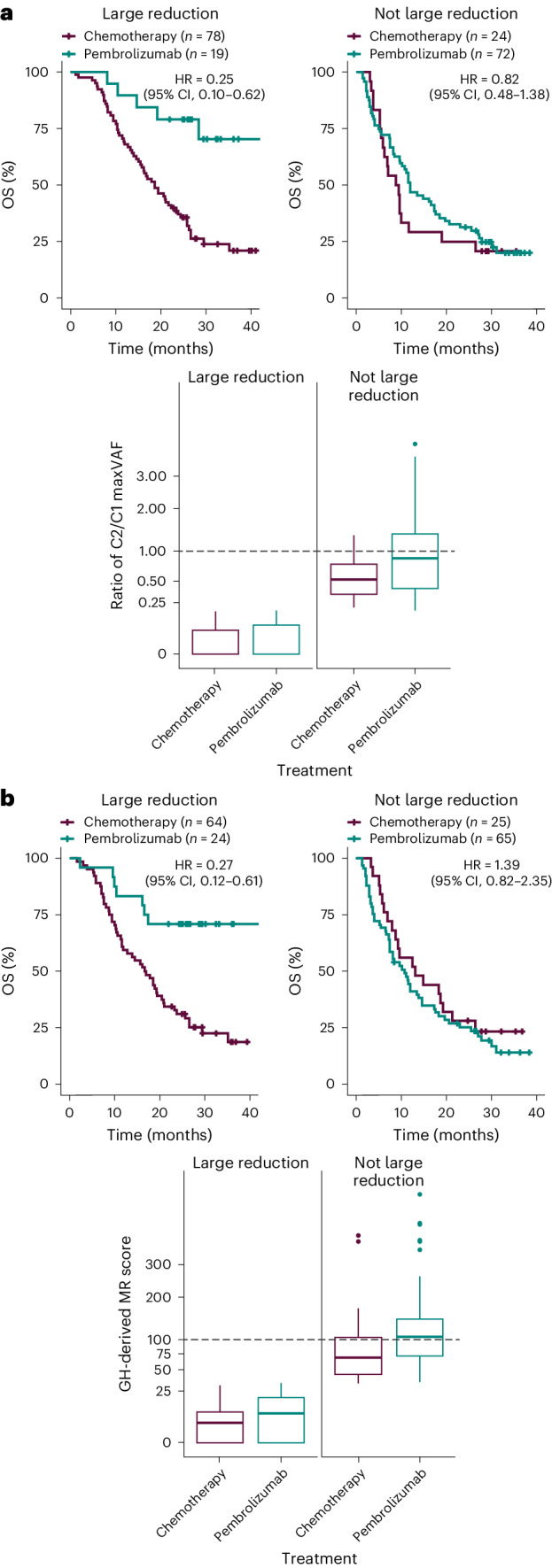
OS for pembrolizumab versus chemotherapy by large and not large reductions of C2/C1. **a**,**b**, Kaplan–Meier estimates of OS by C2/C1 tumor-informed maxVAF (**a**) and MR score changes (**b**). Large reduction = C2/C1 ratio below the median; not large = C2/C1 ratio above the median. Median cutoff 0.18 (**a**), 34 (**b**).

**Fig. 5:**
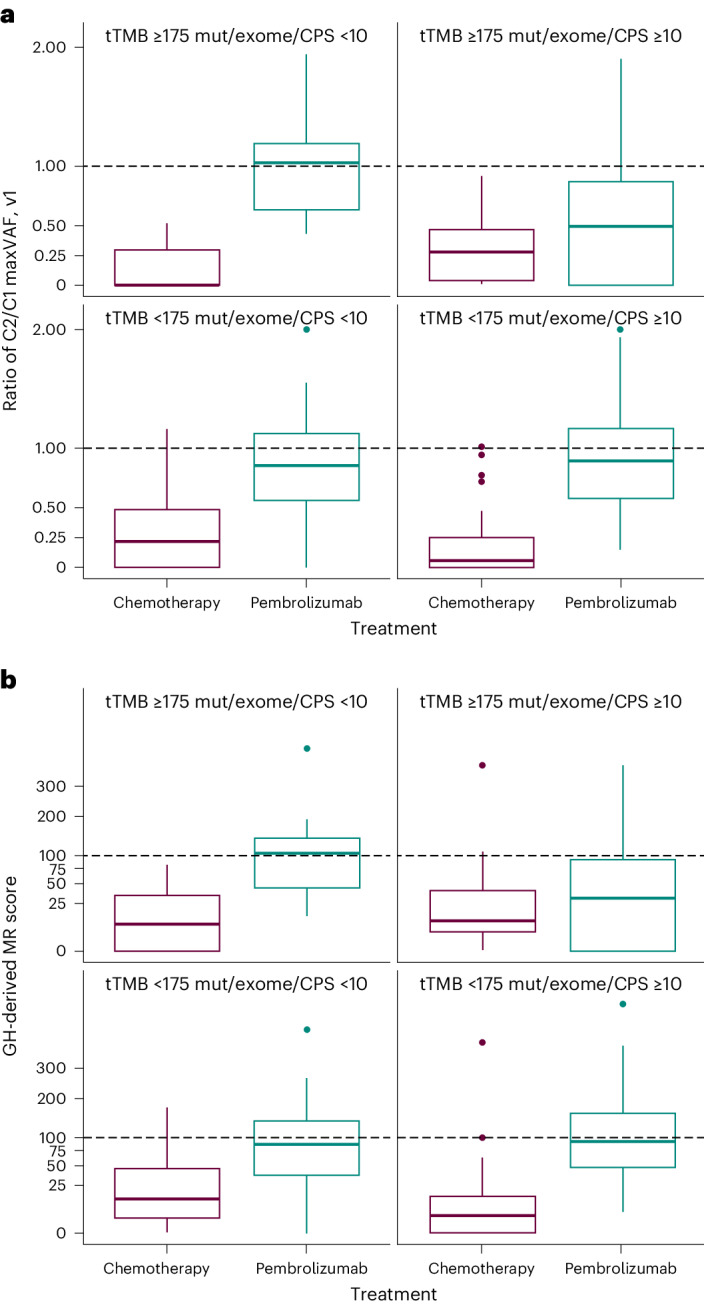
Patient-level C2/C1 changes by tTMB and PD-L1 status and treatment arm. **a**, Tumor-informed maxVAF (pembrolizumab, *n* = 87; chemotherapy, *n* = 102). **b**, MR score changes (pembrolizumab, *n* = 89; chemotherapy, *n* = 89). The center line corresponds to the median and the box is delineated by first and third quartiles. Whiskers extend to any points within 1.5 times the interquartile range, with points lying beyond identified individually as potential outliers.

Notable differences in the OS profile for pembrolizumab versus chemotherapy were observed in patients with similarly large ctDNA reductions based on changes in tumor-informed maxVAF and MR scores (Fig. 4). Evaluation of C2/C1 ctDNA changes by tTMB and PD-L1 status in both the tumor-informed maxVAF and MR score C2 found that the decreases in ctDNA levels in the chemotherapy arm were similar between tTMB and PD-L1 subgroups (Fig. 5). However, in the pembrolizumab arm, the largest reduction in tumor-informed maxVAF and MR score C2/C1 ctDNA changes was observed in the tTMB ≥175 mut/exome and PD-L1 CPS ≥ 10 subgroups (Fig. 5)

## Discussion

There is an ongoing need to better understand the potential role of ctDNA in advanced urothelial carcinoma, including its prognostic value and its role for monitoring long-term efficacy outcomes with different treatments.

Here, we addressed these points in a carefully designed exploratory analysis using a prespecified statistical analysis plan to evaluate patients selected from the pembrolizumab monotherapy and chemotherapy arms of the KEYNOTE-361 study, showing both the prognostic value of ctDNA and distinct treatment effects between chemotherapy and pembrolizumab. ctDNA levels at baseline were found to be robustly associated with clinical outcomes for pembrolizumab but not for chemotherapy. The pembrolizumab association was independent of Eastern Cooperative Oncology Group performance status (ECOG PS), baseline tumor size, and TMB and PD-L1 status. This may be helpful when considering treatment approaches in urothelial carcinoma.

Chemotherapy led to larger overall reductions in ctDNA as well as higher ctDNA clearance compared with pembrolizumab, with the distribution of ctDNA changes correlating with radiological response. When evaluated by the RECIST v.1.1 category, clear treatment-specific differences were observed. The distribution of ctDNA changes decreased when moving from progressive disease to partial response/complete response for both treatment arms; however, the numeric scale associated with any given RECIST v.1.1 category was notably different between the chemotherapy and pembrolizumab arms. These findings may presumably be attributed to the different mechanism of action of pembrolizumab relative to chemotherapy. In addition, these preliminary data suggest that the association between posttreatment ctDNA changes and clinical outcomes, particularly OS, was more pronounced in the pembrolizumab arm relative to the chemotherapy arm, which could be attributable to the durable responses and long-term benefit for patients who do respond to pembrolizumab versus chemotherapy, in which responses are common but short lived. These findings also suggest that the utility of ctDNA as a biomarker may depend on treatment modality and highlight the potential complexity of interpreting ctDNA changes and the connection with long-term survival outcome under certain combination therapies. Results from this analysis reveal important differences in the dynamics of ctDNA beyond PD-L1 expression and TMB and its relationship with clinical outcomes that may reflect the differing mechanisms of action of the treatment and the effects on the dynamics of tumor growth and tumor cell killing.

Strong associations between TMB and clinical outcomes (objective response rate (*P* < 0.001), PFS (*P* < 0.001) and OS (*P* = 0.007)) were observed in patients with advanced urothelial carcinoma treated with pembrolizumab in an exploratory analysis of KEYNOTE-361 (ref. ^24^). These results demonstrated that the associations between ctDNA changes and clinical outcomes observed in the pembrolizumab arm remained statistically significant after adjustment for tTMB and PD-L1 status of the tumor. Furthermore, evaluation of ctDNA changes by tumor tTMB and PD-L1 status shows findings consistent with other posttreatment measures of efficacy (BOR, PFS, OS; that is, differential outcomes with chemotherapy and pembrolizumab according to key tumor biology).

An important question evaluated by this exploratory analysis was whether ctDNA changes provide orthogonal explanatory value for long-term clinical outcomes beyond radiographic tumor size change data. Testing in joint models did not confirm ctDNA changes contained independent value for predicting OS when adjusting for BOR or early percentage change in tumor size. Predictive value for PFS remained after adjusting for BOR. However, early ctDNA changes were assessed at the beginning of treatment C2 (3 weeks), whereas the response by RECIST v.1.1 and tumor size change data came from radiographic evaluations capturing efficacy impact later in time, starting at 9 weeks. In addition, this analysis compared radiological assessment with two ctDNA measurements, as serial ctDNA analyses were not performed. Previous work has shown ctDNA relapse predated radiological relapse in the adjuvant setting^20^. In patients with metastatic gastrointestinal cancer, serial ctDNA monitoring was shown to provide a predictive value of clinical benefit before radiographic assessments^26^. ctDNA changes at 2 weeks did not show a significant correlation with response; however, at 4 weeks, patients with partial responses had a higher decrease in median ctDNA (98.0%) compared with patients with progressive disease (49.0%; *P* < 0.0001)^26^. A similar degree of significance was maintained at 8 weeks^26^. In patients with metastatic breast cancer, serial ctDNA monitoring allowed for the detection of metastasis with an average lead time of 11 months over clinical detection^27^. Serial ctDNA may conceivably be useful for patients in which RECIST v.1.1 evaluation is not helpful, such as in patients with nonmeasurable disease, mixed response or pseudoprogression.

In the current analysis, short-term reductions in ctDNA were not treatment-independent surrogates for clinical outcomes, as similar distributions of ctDNA reductions for pembrolizumab and chemotherapy were found to coexist with notable between-treatment differences in their OS distributions. The group of patients in the pembrolizumab arm with large ctDNA reductions showed a more favorable OS profile relative to patients in the chemotherapy arm who achieved large ctDNA reductions. The use of median change seen across both treatment groups was prespecified in the analysis plan as a cutoff but is solely for illustration. Our findings suggest any framing of cutoffs on ctDNA changes for clinical use as, say, prognostic markers for longer-term patient outcome would have to follow a treatment-specific scale germane to the mechanism of action and line of therapy. There is probably no absolute ctDNA change that can be interpreted independent of such context.

The correlation between pretreatment tumor burden and pretreatment ctDNA levels was modest overall, and the tumor-informed metric shows a fairly broad representation of baseline tumor burden for patients who are all ctDNA negative. Physical and biological factors (for example, lesion location) may influence ctDNA levels and detectability.

Given the different ctDNA testing platforms (tumor-informed versus uninformed, panel-based approach) that are available and have been used in different disease stages of urothelial carcinoma, we also evaluated four different quantitative approaches to explore the relationship of these approaches to clinical outcomes. The tumor-informed version of the ctDNA metric showed the strongest associations with clinical outcomes and was the only version to show consistently significant associations in the chemotherapy arm. This approach tracks variants only found in pretreatment tissue WES and thus reduces the chance of false-positive variant detection in ctDNA, resulting in higher specificity. Tumor-uninformed approaches are more cost-effective because they are designed with a fixed panel of genes but have reduced specificity compared with tumor-informed approaches. Additionally, the simpler uninformed approach may have more utility in an immunotherapy setting than for chemotherapy, and thus may be useful in a subset of patients, such as patients with bone-only disease where standard radiology is unhelpful. Our results show consistent associations with clinical outcomes in the pembrolizumab arm for tumor-uninformed metrics and may indicate that this method has a more compelling application when studying immunotherapies than chemotherapies or combinations with chemotherapies, potentially simplifying ctDNA analysis in the future. The MR assay used in this analysis is a tumor-uninformed approach that uses plasma-only ctDNA and can provide real-time genomic profiling of patients in the clinic^28^. The approach described herein using a mutation-based panel for ctDNA testing is relevant for the advanced and metastatic disease setting such as that in the present study, where ctDNA burden is generally not limiting for detection, with panels with limit of detections in the 0.1–2% range. However, for early-stage disease where nonmeasurable disease is expected, the ctDNA fraction of cell-free DNA is generally <0.1%, thus ctDNA detection methods using more sensitive assays will be necessary.

Personalized tumor-informed tests are validated tests that have been designed and optimized to detect very low levels of ctDNA, making it particularly relevant for use in the early-stage disease setting for the detection of molecular residual disease (and, even further, in the postsurgery setting where resected tumor samples should offer ample material for WES to support design of the personalized ctDNA assay). However, such assay sensitivity may not be necessary in metastatic disease, particularly in a well-shedding tumor type such as bladder cancer. In the present analysis, only a small proportion of patients did not have detectable pretreatment ctDNA using GuardantOMNI. In addition, as the availability, quality and/or quantity of tumor tissue and/or resulting extracted DNA are by far the main reasons for technical failure of tumor-informed ctDNA assays, especially in late-stage disease where tumor tissue can be limited to what is remaining from a diagnostic biopsy, we were particularly interested in assessing the performance of a tumor-independent ctDNA assay in this setting. We used multiple methods of ctDNA analysis to robustly test the hypothesis that immunotherapy and chemotherapy have distinct patterns of ctDNA response. This included an approach of panel-based analysis informed by tumor WES to improve accuracy. Hybrid approaches have been explored previously to improve accuracy, and integrating platforms has been used previously with success^29^. As a result, attempting to improve accuracy of panel-based approaches, which can be hindered by false-positive results, is a worthy endeavor. Given the availability of the WES data, we found this accessible for our investigation.

This ctDNA substudy was designed as a retrospective scientific inquiry aimed at understanding ctDNA, as both a pre- and posttreatment biomarker, under the distinct treatment mechanisms of immunotherapy and chemotherapy, with objectives and a statistical analysis plan prepared before connecting ctDNA data to clinical outcome. Although we strategically aimed to select a representative set of patients for this substudy, our findings are limited by a moderately sized sample from KEYNOTE-361, the retrospective nature of the analysis and that the analysis occurred only in two of the three study arms. In particular, trends in subgroups created by segregating multiple biomarkers should be interpreted with caution and larger prospective studies designed to compare ctDNA clearance patterns in the context of different treatments are needed to confirm the results. Combination therapy was not evaluated, under the concern that the inability to discern which patients are responding to which treatment mechanisms may confound interpretation. Additionally, only one follow-up time point (3 weeks) was evaluated, which is a potential shortcoming. Finally, further serial ctDNA analysis may yield insight into the impact of ctDNA clearance on monitoring treatment responses, which is an area for future research. At this time, validating analyses are required to confirm the clinical utility of ctDNA. An additional question is whether the disease burden captured by the complement of the tumor-informed mutations is being modulated differentially according to treatment. Results from this analysis add to an increasing body of evidence that in the future may enable clinicians to incorporate molecular parameters such as ctDNA into routine oncology care and allow for more appropriate selection of patients likely to benefit from specific treatment modalities.

## Methods

### Study design, patients and treatment

KEYNOTE-361 (NCT02853305) was a randomized, open-label, phase 3 trial conducted across 201 medical centers globally. Details of the trial design and the eligibility criteria have been published^23^. Key eligibility criteria included patients aged ≥18 years with previously untreated locally advanced, unresectable or metastatic urothelial carcinoma; an ECOG PS score of 0 to 2; and one or more measurable lesions per RECIST v.1.1 by investigator assessment. Sex of participants was determined based on self-report. The study protocol and all amendments were approved by the institutional review board or ethics committee at each participating institution. The study was conducted in accordance with the protocol, its amendments and the ethical principles originating from the Declaration of Helsinki and Good Clinical Practice guidelines. Written informed consent was provided by all patients before enrollment.

Patients were randomly assigned 1:1:1 to receive pembrolizumab 200 mg intravenously every 3 weeks for ≤35 cycles (~2 years) plus platinum-based chemotherapy (≤6 cycles of gemcitabine 1,000 mg m^−2^ on day 1 and day 8 every 3 weeks plus investigator’s choice of either cisplatin 70 mg m^−2^ every 3 weeks or carboplatin area under the concentration curve (AUC) 5 mg ml^−1^ min^−1^ intravenously on day 1 of each 3-week cycle), pembrolizumab monotherapy or platinum-based chemotherapy.

This exploratory ctDNA substudy of the KEYNOTE-361 study was conducted in a subset of patients selected from the pembrolizumab and chemotherapy alone arms; ctDNA was not assessed in the combination arm. We were interested in assessing the performance of a tumor-independent ctDNA assay in the metastatic setting to determine whether a tumor-independent approach would have meaningful relationships with clinical outcomes. To optimize the relationship between a panel-based ctDNA test and clinical outcomes, we used several different metrics to calculate ctDNA levels, including maxVAF, meanVAF and a proprietary MR score provided by GH with their GuardantOMNI assay output (the latter metric specifically quantifies changes in ctDNA levels, not baseline levels, so was included only in the change-from-baseline analyses). In addition, we wanted to investigate whether the relationship with clinical outcomes observed using the tumor-independent metrics would be similar to a tumor-informed approach, which was simulated in the present study by using already available tumor tissue and normal WES data from these patients to select a subset of the panel-identified variants (those identified in the tissue WES) to define ctDNA levels. Hence, we designed this ctDNA analysis to use patients with available tissue and normal WES data to evaluate whether the addition of tumor mutation and normal variant information to the panel-based approach would improve the relationship between ctDNA and clinical outcomes and to be able to understand whether tissue TMB is predictor of the magnitude of ctDNA changes under immunotherapy. The overarching aims were to determine whether a set of baseline and change-from-baseline ctDNA metrics were associated with clinical outcomes and to evaluate the patterns of ctDNA response under treatment with immunotherapy (that is, pembrolizumab) versus chemotherapy.

### Outcomes and assessments

BOR of complete or partial response was evaluated per RECIST v.1.1 as assessed by blinded independent central review (BICR). PFS was defined as the time from start of treatment to first documented evidence of disease progression per RECIST v.1.1 as assessed by investigator or death from any cause (whichever occurred first). OS was defined as the time from start of treatment to death from any cause.

ctDNA levels were assessed using the next-generation-sequencing-based GuardantOMNI assay. Descriptive evaluation of the ctDNA mutations from the pembrolizumab and chemotherapy arm are outlined in Extended Data Fig. 2. Four metrics monitoring change in ctDNA levels were evaluated using the ratio of on treatment C2 ctDNA levels compared with the pretreatment cycle (C1).

- Tumor-informed maxVAF, which used paired tissue and matched normal WES to filter putative somatic variants in ctDNA via confirmation from tissue WES;
- Tumor-uninformed maxVAF, which did not use paired WES to filter putative somatic variants in ctDNA;
- Tumor-uninformed meanVAF, which was the average of VAF for somatic mutations restricting attention to those that occurred at C1 (so later variants that might be cleared posttreatment would be included as zeros in the average); and
- GH MR score, which can quantify only ctDNA change (that is, cannot be derived at baseline).

As the GH MR score was evaluable on a smaller number of participants and is not meaningful at baseline, and tumor-informed maxVAF was correlated strongly with tumor-informed meanVAF, the evaluation of baseline (pretreatment) ctDNA levels in association with clinical outcome focused two metrics at C1: tumor-informed maxVAF and tumor-uninformed maxVAF. Summary statistics for each metric at baseline are in the supplement (Supplementary Fig. 3). Additionally, as tumor-informed maxVAF and GH MR scores had the highest fidelity associated with long-term outcomes, they were evaluated further. Median cutoffs on ctDNA changes were used to segregate ctDNA reductions based on whether the C2/C1 ratio was greater or smaller than the median (median was defined across arms and for each ctDNA metric) for the purpose of illustrating qualitative patterns in long-term clinical outcomes for pembrolizumab versus chemotherapy in patients with similar levels of ctDNA changes posttreatment.

Primary prespecified objectives for this exploratory ctDNA analysis were to determine whether baseline or on treatment changes in ctDNA levels, as captured by these metrics, were associated with clinical outcomes (BOR, PFS and OS). Secondary objectives were to evaluate whether baseline and change-from-baseline ctDNA metrics were associated independently with clinical outcomes in models adjusted for biomarker subgroup, other baseline prognostic factors (baseline tumor burden and ECOG PS), and radiographic response by RECIST v.1.1. Investigating ctDNA changes from C1 to C2 by key tumor microenvironment biomarkers (tTMB <175 mut/exome versus ≥175 mut/exome, and PD-L1 CPS < 10 versus CPS ≥ 10 subgroups) separately by treatment arm was another key secondary objective of this exploratory analysis.

Tumor tissue and normal (blood cell) WES was performed as previously described^30^. In brief, WES was performed by using formalin-fixed paraffin-embedded sections of pretreatment tumor samples. After pathology assessment, tissue was scraped from the entire section with a fresh scalpel and transferred to a 1.5-ml tube containing 200 μl of 100% ethanol. DNA was isolated using the QIAamp DNA FFPE Tissue Kit (Qiagen). Thereafter, tumor DNA was quantitated using the Qubit assay (Invitrogen) and quality was assessed using the QuantideX qPCR DNA QC Assay (Asuragen). Matched normal DNA was extracted from whole blood collected in a PAXgene Blood DNA Tube (Qiagen) at clinical sites and stored at −20 °C or −70 °C/−80 °C until processed in an approved central laboratory identified by the sponsor. The Chemagic STAR DNA Blood Kit (PerkinElmer) run on either a Hamilton Chemagic STAR or PerkinElmer Chemagic 360 automated instrument was used to extract DNA in a final volume of 500 μl or 1.0 ml extracted DNA was subjected to volume and concentration determination and ultraviolet and visible spectral analysis to assess purity. WES was performed using ACE Cancer Exome (Personalis) with average coverage X (range Y–Z). WES reads were aligned to reference human genome GRCh37 by using bwa mem followed by preprocessing steps including duplicate marking, indel realignment and base recalibration with Picard (v.1.114) and GATK (Genome Analysis Toolkit, v.2) to generate analysis-ready BAM files. MuTect was used to generate somatic single nucleotide variant (SNV) calls using default parameters by comparing BAM files from tumor and matched normal samples. MuTect-called SNVs present in the Single Nucleotide Polymorphism Database (dbSNP, v.141) but not in the Catalogue of Somatic Mutations in Cancer (COSMIC, v.68) were filtered out. SNVs with mutant reads of fewer than four in tumor samples were also eliminated. TMB for a patient was defined as the sum of somatic nonsynonymous SNVs that passed all the filters described. There were 4,185 somatic mutations detected in the Guardant assay, 72,832 somatic mutations detected in WES and 3,836 somatic mutations detected in WES that overlapped with Guardant regions. Plasma ctDNA was evaluated using the GH GuardantOMNI assay and has been described previously^31^. This hybridization next-generation sequencing ctDNA detection assay is panel-based, tests for 500 genes and has a limit of detection of 0.24% to 0.6% VAF for 30-ng input cfDNA for SNV detection. ctDNA was collected at predose before the first cycle of therapy (D1, ±3 days) and 3 weeks thereafter for the postbaseline assessment, (D22 (±3 days) collection, coinciding with the C2 of therapy. PD-L1 expression was determined using PD-L1 IHC 22C3 pharmDx (Agilent). PD-L1 CPS was calculated as the number of PD-L1–staining cells (tumor cells, lymphocytes and macrophages) divided by the total number of viable tumor cells, multiplied by 100.

### Statistical analysis

This exploratory analysis was conducted per a prespecified statistical analysis plan. The eligible analysis population was required to have both WES data and a baseline ctDNA sample available for sequencing. Before sample selection for this analysis, power calculations were performed to determine the sample size needed to ensure sufficient power for testing associations with clinical outcomes.

The priority objective used to determine sample size was testing for an association between pretreatment ctDNA levels and OS. Bootstrap sampling of data from preliminary work conducted on trials outside of KEYNOTE-361 across different settings of sample sizes suggested a design targeting 125 patients per arm could achieve 80% power after correcting for multiple testing (assuming at least two tests on pretreatment ctDNA metrics and a Bonferroni correction 0.05/2 = 0.025, one sided). Patient samples were selected using a stratified sampling procedure such that the proportions of patients in each of the four tTMB and PD-L1 CPS subgroups matched those from the entire analysis-eligible population (acknowledging the relationship between tTMB and PD-L1 CPS subgroups and clinical outcomes observed in the KEYNOTE-361 exploratory biomarker analyses)^24^. An additional 5% of patients were sampled to account for potential quality control failures. In total, 131 patients from the chemotherapy arm and 132 patients from the pembrolizumab arm were sent for ctDNA profiling (although not all specimens sent for profiling were successfully assayed).

OS and PFS Kaplan–Meier curves, both overall and in tTMB and PD-L1 CPS subgroups, were examined visually to ensure the selected patients were representative of both the intention-to-treat and WES-available populations in terms of clinical outcomes (Supplementary Figs. 4 and 5).

The testing of baseline and change-from-baseline ctDNA metrics for their associations with clinical outcomes were all prespecified in the statistical analysis plan, as well as the testing to determine the independent explanatory value of the ctDNA metrics beyond the predictive information contained in TMB/PD-L1 status and in radiographic measures of tumor response. Associations with BOR were evaluated separately in each arm using logistic regression. Associations with PFS and OS was evaluated using Cox proportional hazards regression. Both logistic regression and Cox regression models were adjusted for ECOG PS and robustness of findings was further assessed by adjusting for baseline tumor size, tTMB and PD-L1 status and the information contained in radiographic measures of response (BOR by RECIST v.1.1, or percentage tumor shrinkage at 9 weeks). Models adjusting for tTMB/PD-L1 status used a four-level factor created by the TMB cutoff of 175 mut/exome and PD-L1 CPS cutoff of ten. The Kaplan–Meier method was used for generating survival curves and Cox models were used to estimate between-arm hazard ratios and corresponding 95% CIs.

Multiplicity-adjusted statistical significance testing was conducted using the Hochberg step-up procedure for family-wise-error control^32^. Testing penalties were applied across the number of biomarkers tested (four posttreatment monitoring metrics tested and two baseline metrics tested, treated as separate families) with an adjusted significance level prespecified at *α* = 0.05. No multiple testing penalty was applied for the different clinical outcomes tested, rather consistency of testing conclusions across clinical outcomes was emphasized. The statistical significance was based on *P* values which concurrently assess the strength of the association and the number of events. Differences related to the associations between ctDNA and outcomes in the two arms are based on *P* values.

### Reporting summary

Further information on research design is available in the Nature Portfolio Reporting Summary linked to this article.

## Online content

Any methods, additional references, Nature Portfolio reporting summaries, source data, extended data, supplementary information, acknowledgements, peer review information; details of author contributions and competing interests; and statements of data and code availability are available at 10.1038/s41591-024-03091-7.

## Supplementary information

Supplementary Information Supplementary Figs. 1–5 and Table 1.

Reporting Summary

## Figures and Tables

**Extended Data Table 1:**
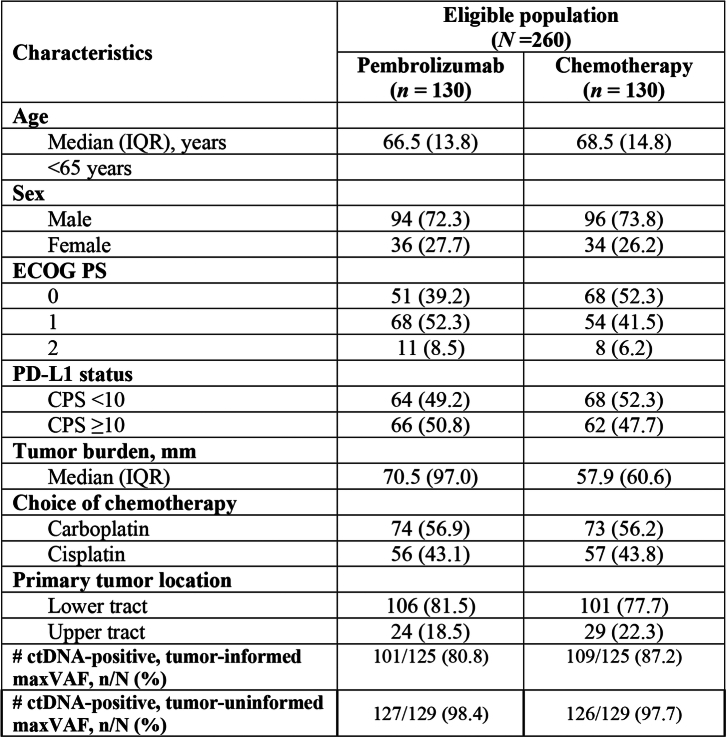
Baseline characteristics for the analysis-eligible population

Data are n (%) except otherwise noted. CPS, combined positive score; ctDNA, circulating tumor DNA; ECOG PS, Easter Cooperative Oncology Group performance status; IQR, interquartile range; maxVAF, maximum variant allele frequency; PD-L1, programmed death ligand 1.

**Extended Data Table 2:**
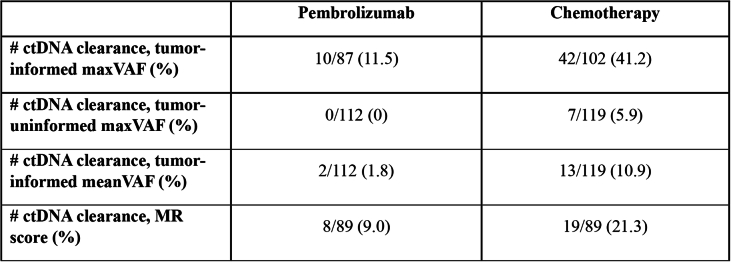
Percentage of patients with ctDNA clearance at C2^a^

C2, cycle 2; ctDNA, circulating tumor DNA; maxVAF, maximum variant allele frequency; meanVAF, mean variant allele frequency; MR, molecular response. ^a^Restricted to patients with baseline ctDNA >0.

**Extended Data Table 3:**
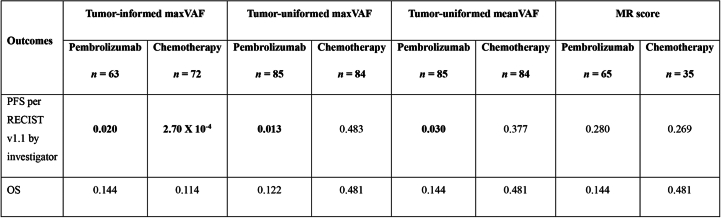
Association between C2/C1 ctDNA change and clinical outcomes after adjustment for tTMB and PD-L1 status and 9-week percentage change in tumor size

Association was evaluated using Cox proportional hazards regression (PFS and OS), with adjustment for ECOG PS. Multiplicity-adjusted P values were calculated. Significance was prespecified at α = 0.05. Bold font indicates significance. Hypothesis: one-sided alternative hypothesis testing for a negative association. C1, cycle 1; C2, cycle 2; ctDNA, circulating tumor DNA; BOR, best overall response; ECOG PS, Eastern Cooperative Oncology Group performance status; maxVAF, maximum variant allele frequency; meanVAF, mean variant allele frequency; MR, molecular response; OS, overall survival; PFS, progression-free survival; RECIST v1.1, Response Evaluation Criteria in Solid Tumors version 1.1; tTMB, tissue tumor mutational burden.

**Extended Data Table 4:**
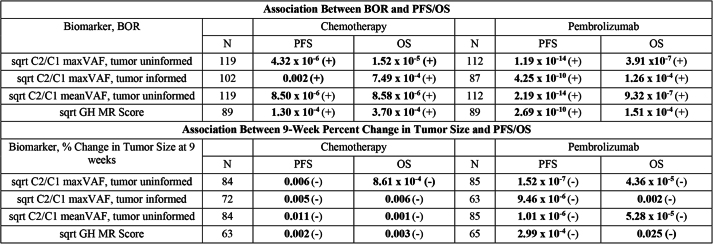
Association between BOR or 9-week percentage change in tumor size (2-sided P value) and PFS/OS in joint models adjusting for the stated C2/C1 ctDNA change metric and tTMB/PD-L1 subgroup

Association was evaluated using Cox proportional hazards regression (PFS and OS). All models include extra covariate(s) of ECOG+TMBCPScat. Models for MaxVAF or MeanVAF additionally adjusted for relevant C1 (baseline) MaxVAF or MeanVAF (square-root transformed). Nominal significance was prespecified at α = 0.05. Bold font indicates significance. BOR hypothesis: nonzero association. +/−: indicating the observed association is positive/negative. C1, cycle 1; C2, cycle 2; ctDNA, circulating tumor DNA; BOR, best overall response; maxVAF, maximum variant allele frequency; meanVAF, mean variant allele frequency; MR, molecular response; OS, overall survival; PFS, progression-free survival; RECIST v1.1, Response Evaluation Criteria in Solid Tumors version 1.1; tTMB, tissue tumor mutational burden.

## Data Availability

Merck Sharp & Dohme LLC, a subsidiary of Merck & Co., Inc. (MSD), is committed to providing qualified scientific researchers access to anonymized data and clinical study reports from the company’s clinical trials for the purpose of conducting legitimate scientific research. MSD is also obligated to protect the rights and privacy of trial participants and, as such, has a procedure in place for evaluating and fulfilling requests for sharing company clinical trial data with qualified external scientific researchers. The MSD data-sharing website (http://engagezone.msd.com/ds_documentation.php) outlines the process and requirements for submitting a data request. Applications will be assessed promptly for completeness and policy compliance. Feasible requests will be reviewed by a committee of MSD subject matter experts to assess the scientific validity of the request and the qualifications of the requestors. In line with data privacy legislation, submitters of approved requests must enter into a standard data-sharing agreement with MSD before data access is granted. Data will be made available for request after product approval in the United States and the European Union or after product development is discontinued. There are circumstances that may prevent MSD from sharing requested data, including country or region-specific regulations. If the request is declined, it will be communicated to the investigator. Access to genetic or exploratory biomarker data requires a detailed, hypothesis-driven statistical analysis plan that is developed collaboratively by the requestor and MSD subject matter experts; after approval of the statistical analysis plan and execution of a data-sharing agreement, MSD will either perform the proposed analyses and share the results with the requestor or will construct biomarker covariates and add them to a file with clinical data that is uploaded to an analysis portal so that the requestor can perform the proposed analyses.
