# Supplementary material for: Pembrolizumab for advanced urothelial carcinoma: exploratory ctDNA biomarker analyses of the KEYNOTE-361 phase 3 trial
Source: Nat Med. 2024 Jun 1;30(9):2508–16. doi: 10.1038/s41591-024-03091-7 (PMC11405267; doi:10.1038/s41591-024-03091-7)

nature medicine

Supplementary information https://doi.org/10.1038/s41591-024-03091-7

Pembrolizumab for advanced urothelial carcinoma: exploratory ctDNA biomarker analyses of the KEYNOTE-361 phase 3 trial

In the format provided by the authors and unedited

Supplementary appendix

Table of Contents

Supplementary Fig. 1. Baseline ctDNA assessment by tumor uninformed maxVAF and association with clinical outcomes (A) Patient-level baseline tumor-uninformed maxVAF by response status and treatment arm (pembrolizumab n = 129; chemotherapy n = 129) and (B) association between baseline ctDNA and clinical outcomes by tumor-uninformed maxVAF. ……………….………………………………………….…….2

Supplementary Table 1. Association between C2/C1 ctDNA change and clinical outcome by tumor-uninformed maxVAF and tumor-uninformed meanVAF…………………………………………………...4

Supplementary Fig. 2. C2/C1 ctDNA assessment for tumor-uninformed maxVAF and tumor-uniformed mean VAF score. Patient-level C2/C1 (A) tumor-uninformed maxVAF (pembrolizumab n = 112; chemotherapy, n = 102) and (B) tumor-uninformed meanVAF (pembrolizumab, n = 112; chemotherapy, n = 119) changes by response status and treatment arm.. ……………………………………………...…….5

Supplementary Fig. 3. Summary statistics for pretreatment C1 metrics at baseline…………………….6

Supplementary Fig. 4. Survival for populations used for the analysis. Kaplan-Meier estimates of (A) PFS and (B) OS in the ITT population, WES-available population, population selected for analysis, and population not selected for analysis……………………………………………………………...…7

Supplementary Fig. 5. Survival for WES-available and analysis population by tTMB and PD-L1 status**.** Kaplan-Meier estimates of (A) PFS and (B) OS in the WES-available population and the population selected for analysis by tTMB and PD-L1 status……………………………………………………...9

Supplementary Fig. 1. Baseline ctDNA assessment by tumor uninformed maxVAF and association with clinical outcomes (A) Patient-level baseline tumor-uninformed maxVAF by response status and treatment arm (pembrolizumab n = 129; chemotherapy n = 129) and (B) association between baseline ctDNA and clinical outcomes by tumor-uninformed maxVAF.

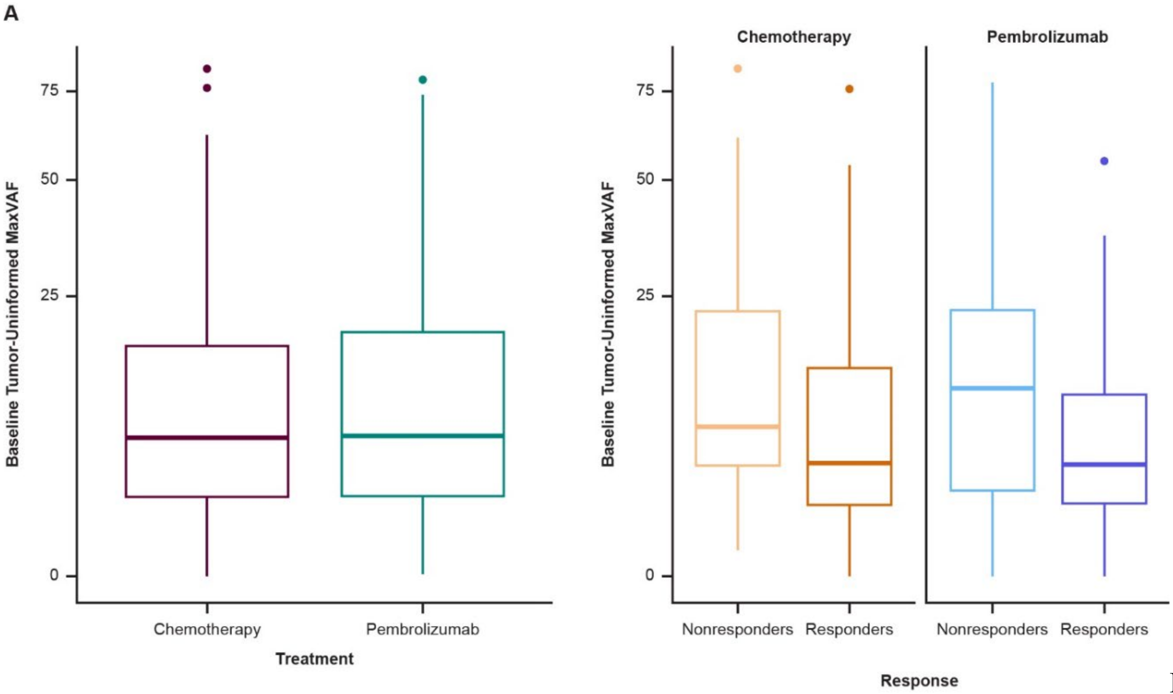

B.

**Outcomes**	**Association between baseline ctDNA and clinical outcomes**		**Association between baseline ctDNA and clinical outcomes after adjustment for baseline tumor size and tTMB/PD-L1 status**	
	Pembrolizumab<br>*n* = 129	Chemotherapy<br>*n* = 129	Pembrolizumab<br>*n* = 121	Chemotherapy<br>*n* = 119
BOR per RECIST v1.1 by BICR	**0.009**	0.172	0.058	0.457
PFS RECIST v1.1 by investigator	**1.42×10^{-5}**	0.060	**0.002**	0.122
OS	**3.50×10^{-6}**	0.088	**3.97×10^{-5}**	0.081

BICR, blinded independent central review; BOR, best overall response; ctDNA, circulating tumor DNA; ECOG PS, Eastern Cooperative Oncology Group performance status; maxVAF, maximum variant allele frequency; OS, overall survival; PD-L1, programmed cell death ligand 1; PFS, progression-free survival; RECIST v1.1, Response Evaluation Criteria in Solid Tumors version 1.1; tTMB, tissue tumor mutational burden.

For Supplementary figure 1A, the center line corresponds to the median and the box is delineated by 1st and 3rd quartiles. Whiskers extend to any points within 1.5 times the interquartile range, with points lying beyond identified individually as potential outliers.

Association was evaluated using logistic regression (BOR) and Cox proportional hazards regression (PFS, and OS), with adjustment for ECOG PS. Multiplicity-adjusted *P* values were calculated. Significance was prespecified at $\alpha = 0.05$. Bold font indicates significance. Hypothesis: negative association.

Supplementary Table 1. Association between C2/C1 ctDNA change and clinical outcome by tumor-uninformed maxVAF and tumor-uninformed meanVAF.

	**Tumor-uninformed maxVAF**				**Tumor-uninformed meanVAF**			
**Outcomes**	**Association between C2/C1 ctDNA change and clinical outcomes**		**Association between C2/C1 ctDNA change and clinical outcomes after adjustment for tTMB and PD-L1 status**		**Association between C2/C1 ctDNA change and clinical outcomes**		**Association between C2/C1 ctDNA change and clinical outcomes after adjustment for tTMB and PD-L1 status**	
	Pembrolizumab *n* = 112	Chemotherapy *n* = 102	Pembrolizumab *n* = 112	Chemotherapy *n* = 119	Pembrolizumab *n* = 112	Chemotherapy *n* = 119	Pembrolizumab *n* = 112	Chemotherapy *n* = 119
**BOR per RECIST v1.1 by BICR**	**1.14 x 10^{-4}**	**0.006**	**2.53 x 10^{-4}**	**0.009**	**5.85 x 10^{-5}**	**9.34 x 10^{-4}**	**1.04 x 10^{-4}**	**0.001**
**PFS per RECIST v1.1 by investigator**	**2.80 x 10^{-6}**	0.227	**5.08 x 10^{-5}**	0.237	**3.00 x 10^{-7}**	0.165	**2.20 x 10^{-5}**	0.215
**OS**	**7.07 x 10^{-5}**	0.264	**0.002**	0.272	**2.48 x 10^{-5}**	0.264	**0.001**	0.272

**Association between C2/C1 ctDNA change and clinical outcome after adjustment for TMB and PD-L1 status and BOR**				
	**Tumor-uniformed maxVAF**		**Tumor-uninformed meanVAF**	
**Outcomes**	Pembrolizumab *n* = 112	Chemotherapy *n* = 119	Pembrolizumab *n* = 112	Chemotherapy *n* = 119
**PFS per RECIST v1.1 by investigator**	**0.016**	0.857	**0.016**	0.857
**OS**	0.364	0.823	0.364	0.823

Association was evaluated using logistic regression (BOR) and Cox proportional hazards regression (PFS, OS), with adjustment for ECOG PS. Multiplicity-adjusted *P* values were calculated. Significance was prespecified at α=0.05. Bold font indicates significance. Hypothesis: one-sided alternative hypothesis testing for a negative association. BICR, blinded independent central review; BOR, best overall response; C1, cycle 1; C2, cycle 2; ctDNA, circulating tumor DNA; ECOG PS, Eastern Cooperative Oncology Group performance status; maxVAF, maximum variant allele frequency; meanVAF, mean variant allele frequency; OS, overall survival; PFS, progression-free survival; RECIST v1.1, Response Evaluation Criteria in Solid Tumors version 1.1.; tTMB, tissue tumor mutational burden.

Supplementary Fig. 2. C2/C1 ctDNA assessment for tumor-uninformed maxVAF and tumor-uniformed mean VAF score. Patient-level C2/C1 (A) tumor-uninformed maxVAF (pembrolizumab n = 112; chemotherapy, n = 102) and (B) tumor-uninformed meanVAF (pembrolizumab n = 112; chemotherapy, n = 119) changes by response status and treatment arm.

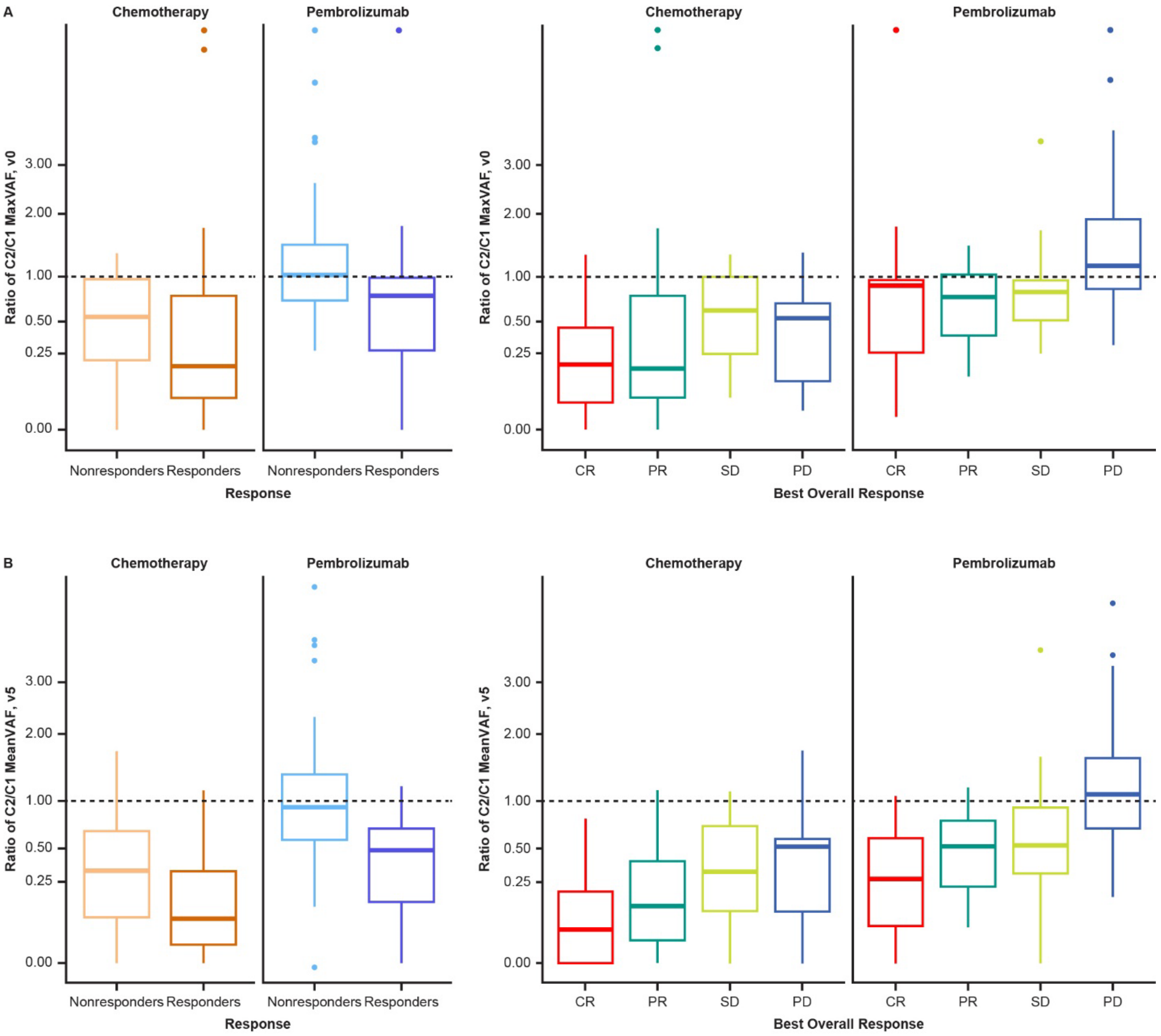

The center line corresponds to the median and the box is delineated by 1st and 3rd quartiles. Whiskers extend to any points within 1.5 times the interquartile range, with points lying beyond identified individually as potential outliers

Supplementary Fig. 3. Summary statistics for pretreatment C1 metrics at baseline.

ctDNA metric	C1 Median (1Q-3Q)	C1 Min, Max
Tumor-uninformed maxVAF	6.13 (2.02-18.8)	0.00, 81.5
Tumor-informed maxVAF	3.84 (0.59-14.5)	0.00, 81.5

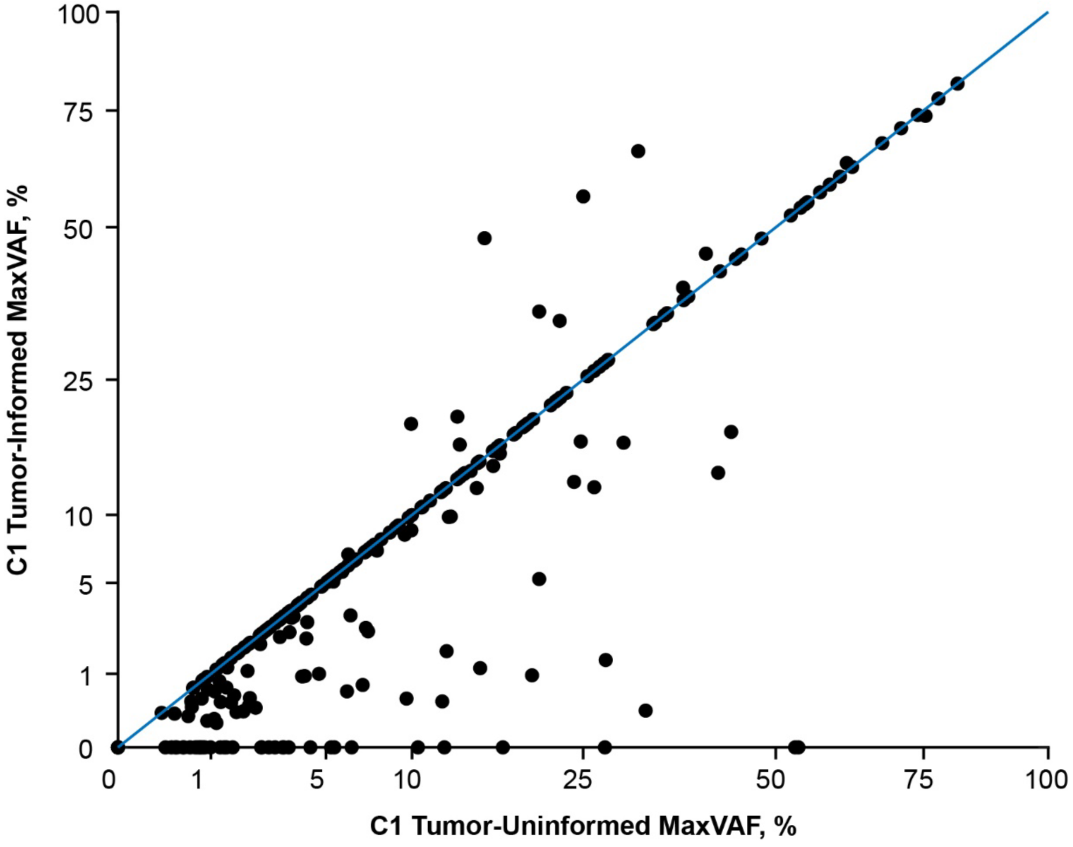

Patients with Guardant Health baseline ctDNA and matched WES tissue data; n = 250. Spearman correlation coefficient r = 0.82 for tumor uninformed maxVAF vs. tumor informed maxVAF.

Supplementary Fig. 4. Survival for populations used for the analysis. Kaplan-Meier estimates of (A) PFS and (B) OS in the ITT population, WES-available population, population selected for analysis, and population not selected for analysis. HR, hazard ratio; ITT, intention to treat; OS, overall survival; PFS, progression-free survival; WES, whole-exome sequencing.

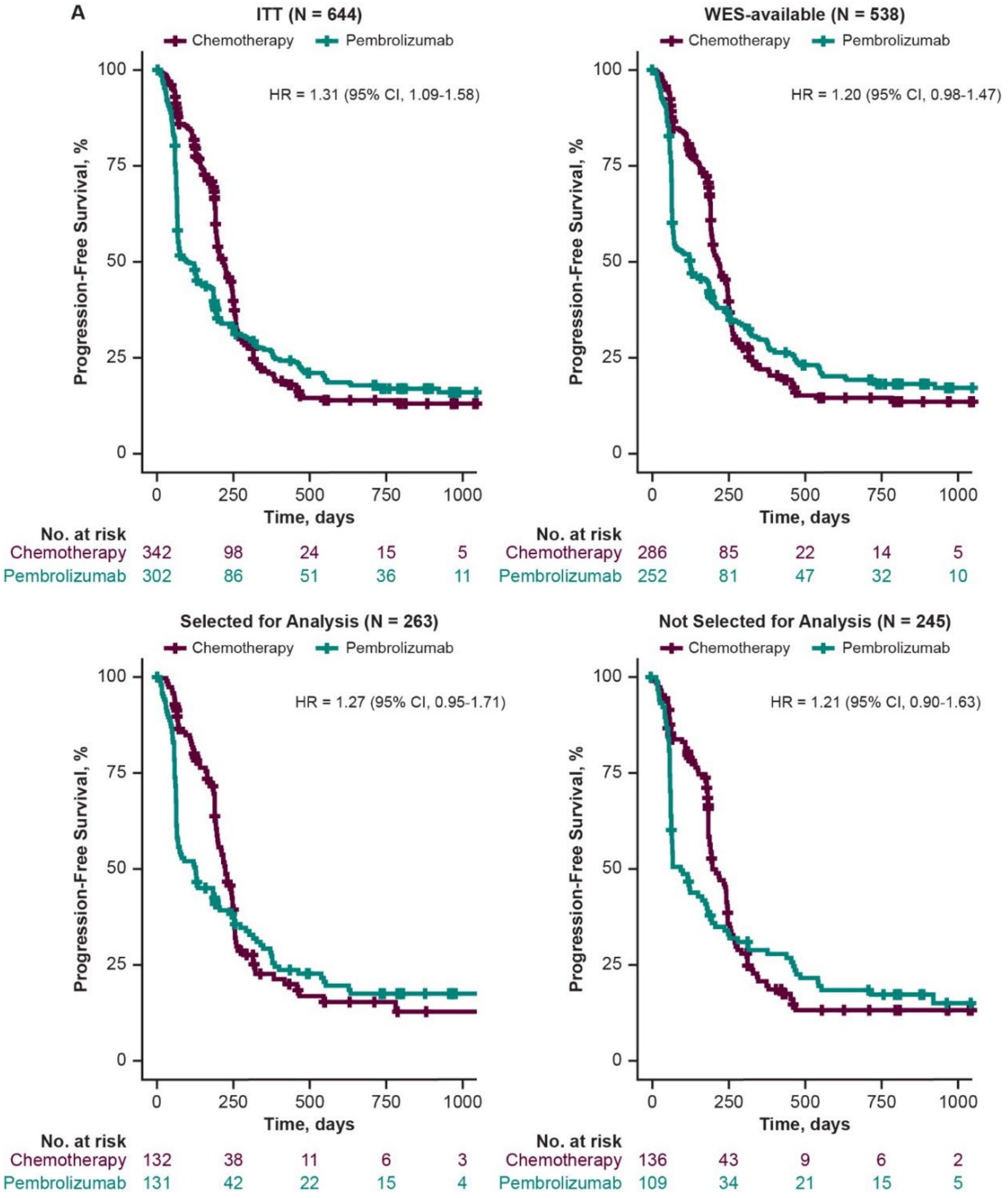

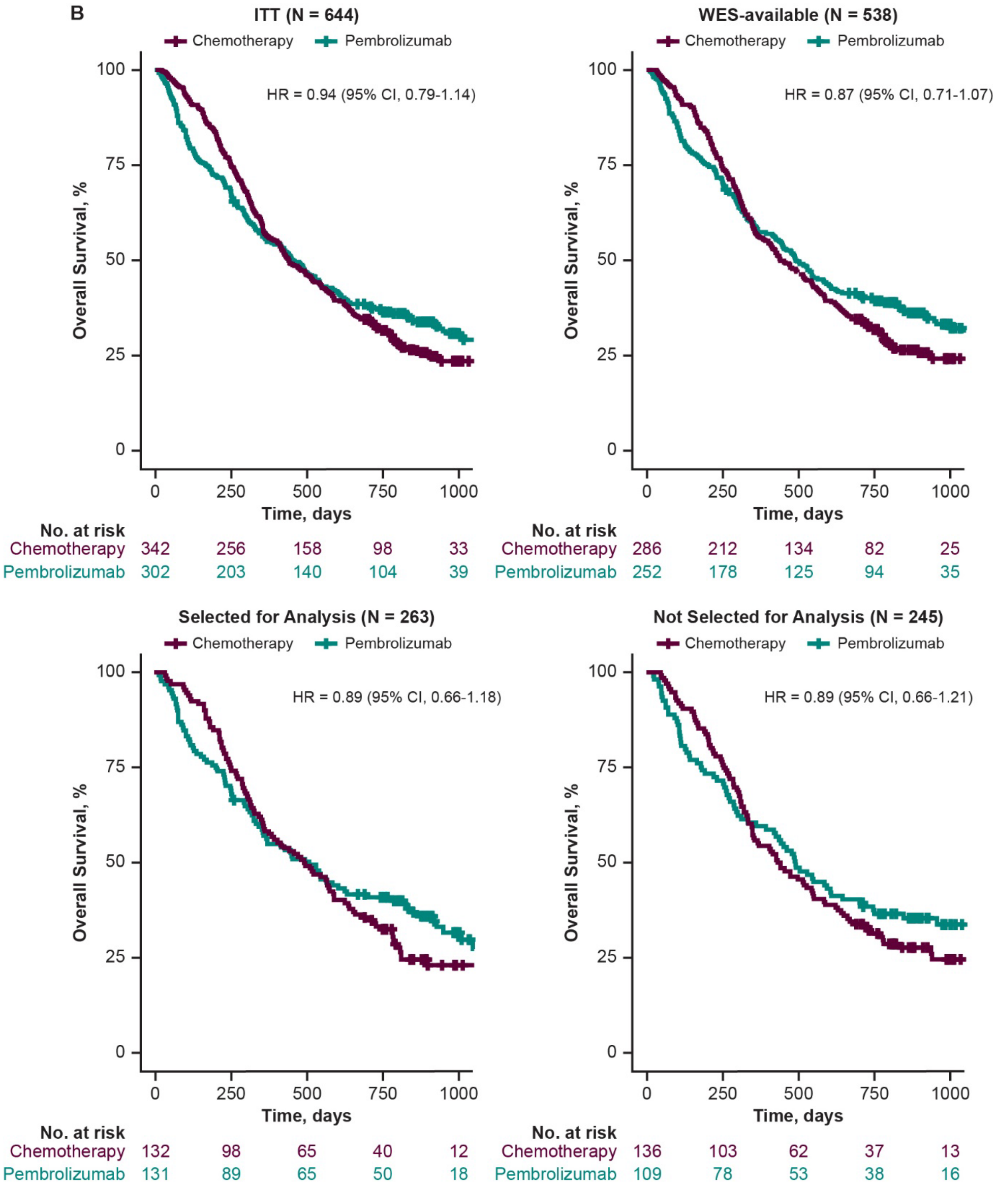
B
ITT (N = 644)
Chemotherapy
Pembrolizumab
HR = 0.94 (95% CI, 0.79-1.14)
Overall Survival, %
100
75
50
25
0
0
250
500
750
1000
Time, days
No. at risk
Chemotherapy 342 256 158 98 33
Pembrolizumab 302 203 140 104 39
WES-available (N = 538)
Chemotherapy
Pembrolizumab
HR = 0.87 (95% CI, 0.71-1.07)
Overall Survival, %
Time, days
No. at risk
Chemotherapy 286 212 134 82 25
Pembrolizumab 252 178 125 94 35
Selected for Analysis (N = 263)
Chemotherapy
Pembrolizumab
HR = 0.89 (95% CI, 0.66-1.18)
Overall Survival, %
Time, days
No. at risk
Chemotherapy 132 98 65 40 12
Pembrolizumab 131 89 65 50 18
Not Selected for Analysis (N = 245)
Chemotherapy
Pembrolizumab
HR = 0.89 (95% CI, 0.66-1.21)
Overall Survival, %
Time, days
No. at risk
Chemotherapy 136 103 62 37 13
Pembrolizumab 109 78 53 38 16

Supplementary Fig. 5. Survival for WES-available and analysis population by tTMB and PD-L1 status. Kaplan-Meier estimates of (A) PFS and (B) OS in the WES-available population and the population selected for analysis by tTMB and PD-L1 status

HR, hazard ratio; OS, overall survival; PD-L1, programmed cell death ligand 1; PFS, progression-free survival; tTMB, tissue tumor mutational burden; WES, whole-exome sequencing.

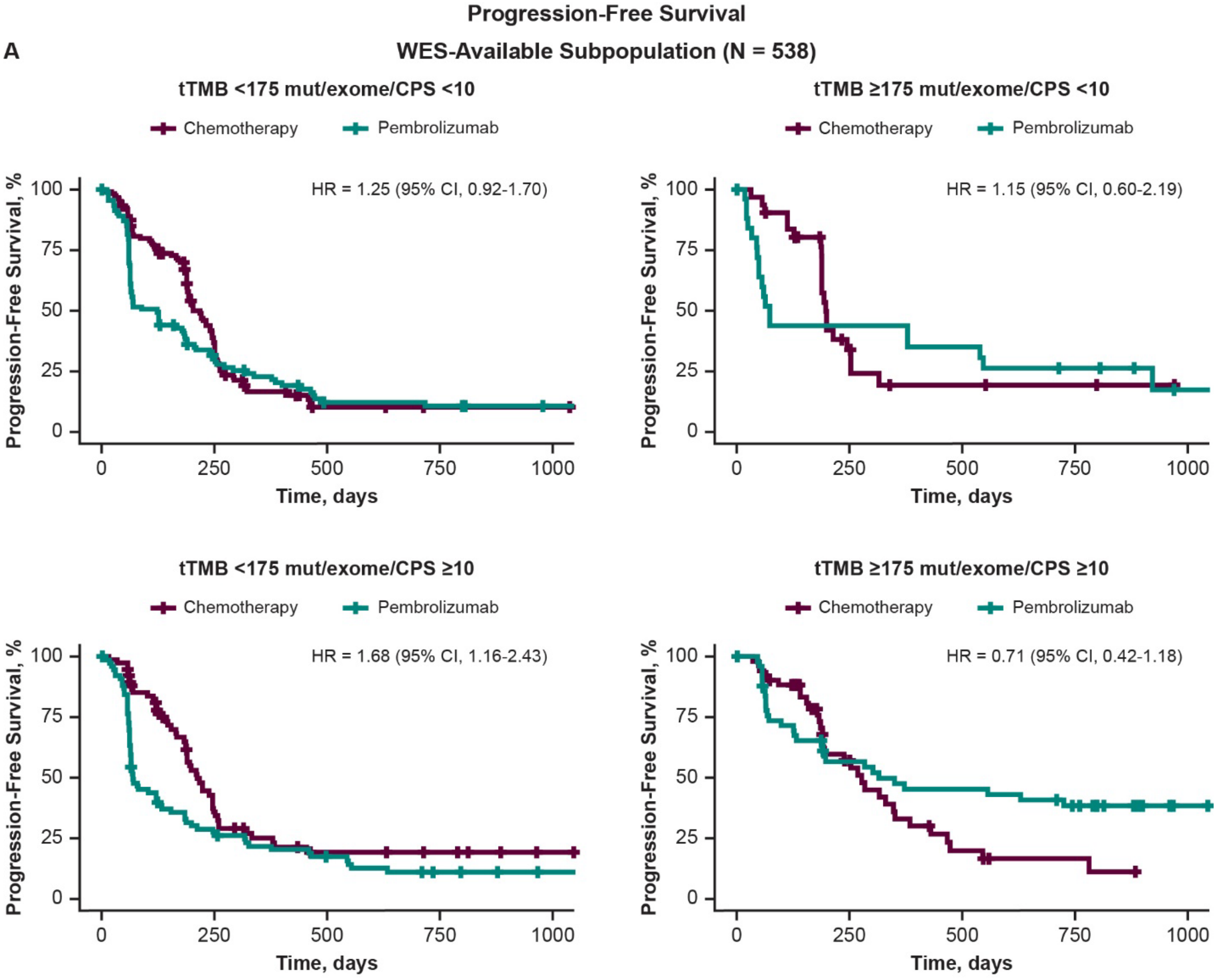

Progression-Free Survival

Analysis Sample (N = 263)

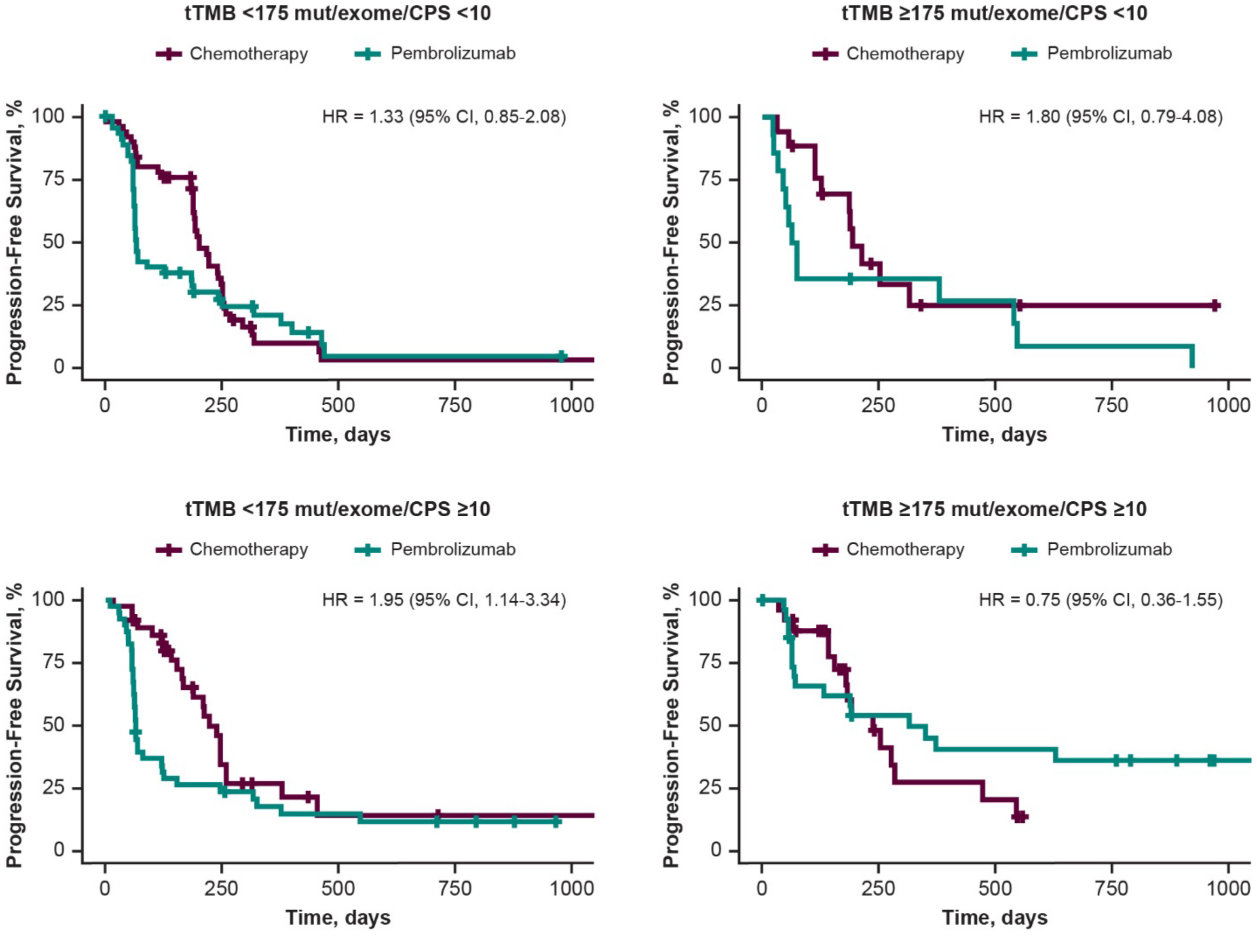

B

Overall Survival

WES-Available Subpopulation (N = 538)

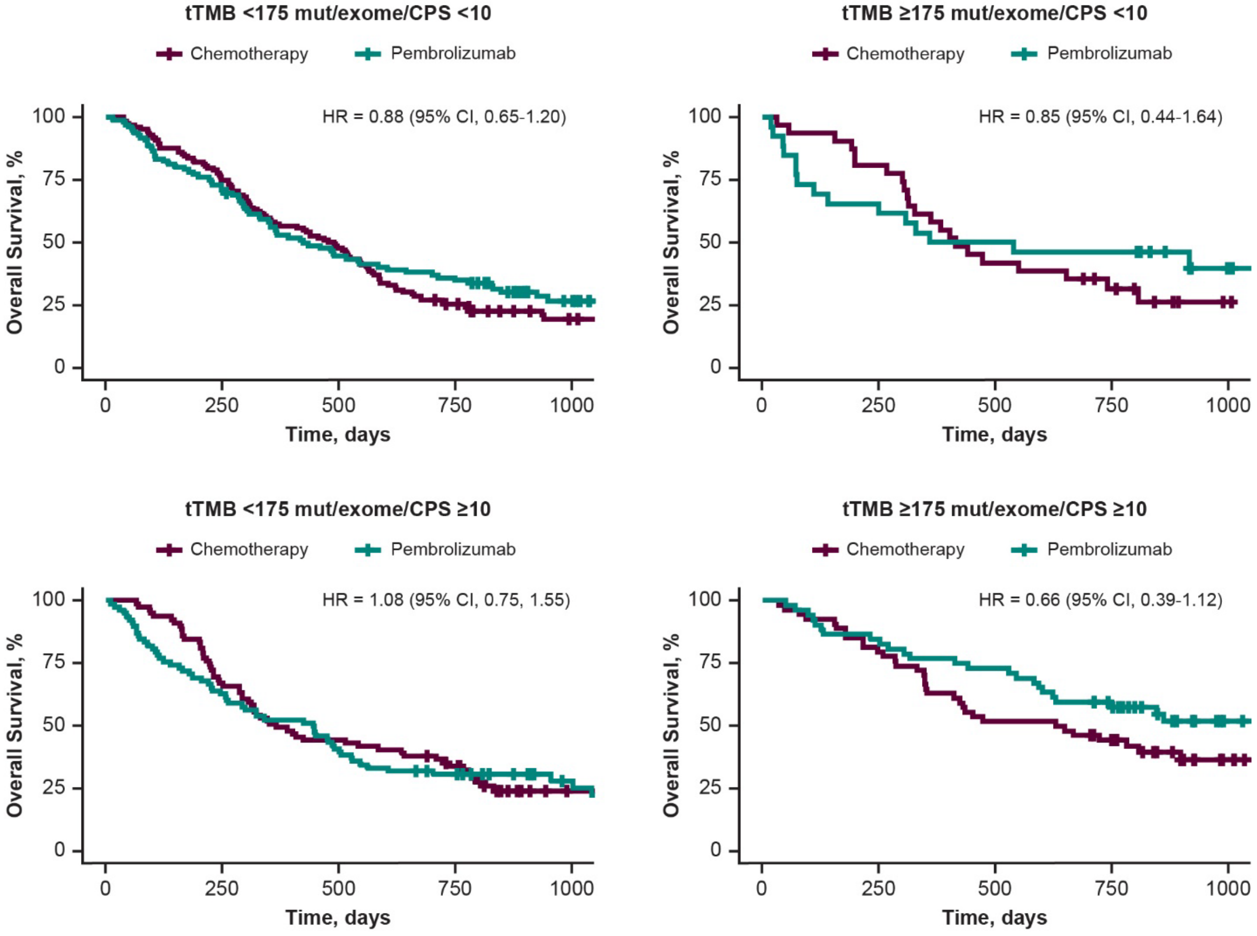

Overall Survival

Analysis Sample (N = 263)

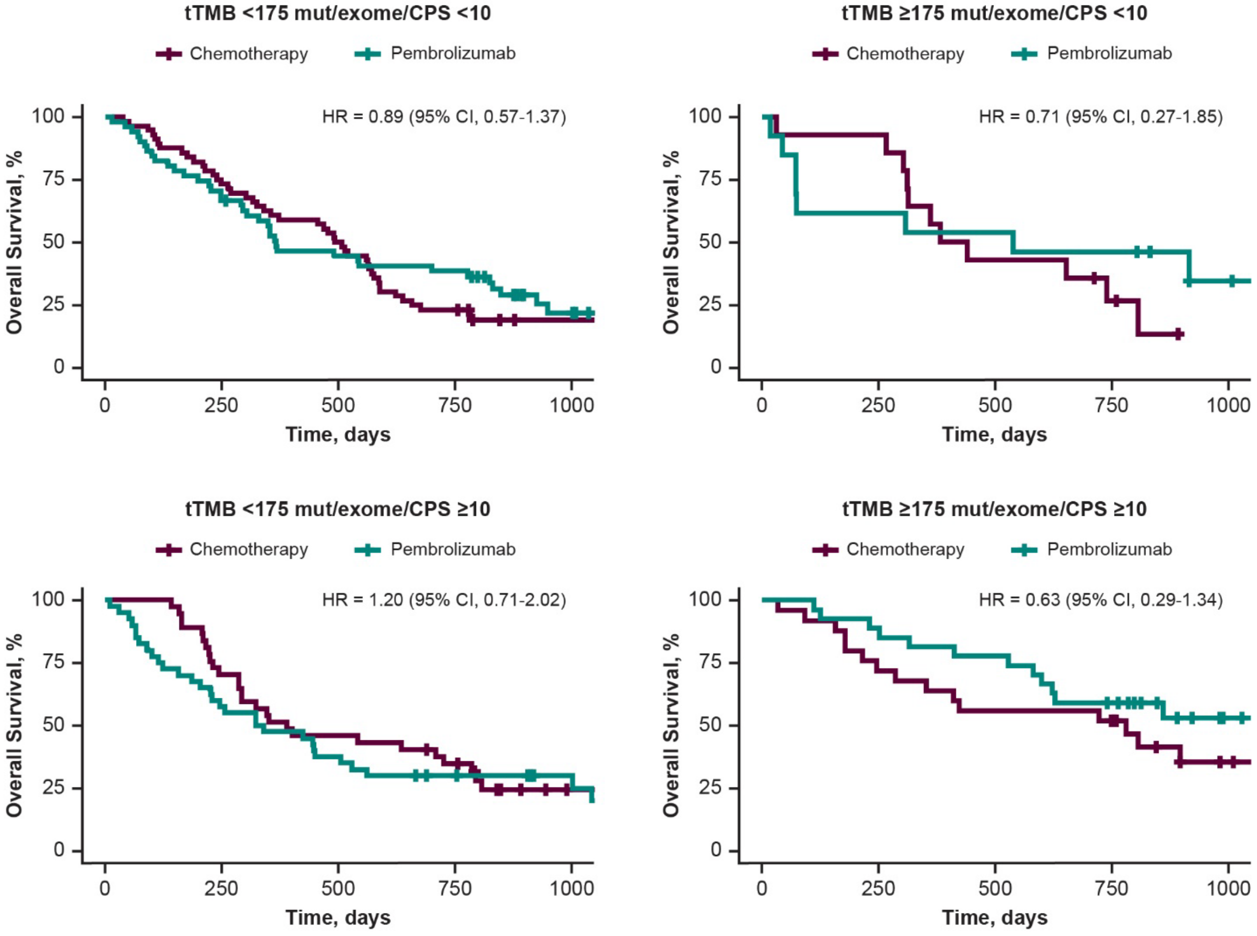

Supplement: Supplementary file 1 — Supplementary Figs. 1–5 and Table 1. [file 41591_2024_3091_MOESM1_ESM.pdf]
