# Supplementary material for: Pembrolizumab for advanced urothelial carcinoma: exploratory ctDNA biomarker analyses of the KEYNOTE-361 phase 3 trial
Source: Nat Med. 2024 Jun 1;30(9):2508–16. doi: 10.1038/s41591-024-03091-7 (PMC11405267; doi:10.1038/s41591-024-03091-7)
Supplement: Supplementary file 2 — Reporting Summary [file 41591_2024_3091_MOESM2_ESM.pdf]

nature portfolio

Corresponding author(s): Thomas Powles

Last updated by author(s): May 21, 2024

Reporting Summary

Nature Portfolio wishes to improve the reproducibility of the work that we publish. This form provides structure for consistency and transparency in reporting. For further information on Nature Portfolio policies, see our Editorial Policies and the Editorial Policy Checklist.

Statistics

For all statistical analyses, confirm that the following items are present in the figure legend, table legend, main text, or Methods section.

n/a	Confirmed	
☐	☒	The exact sample size (*n*) for each experimental group/condition, given as a discrete number and unit of measurement
☒	☐	A statement on whether measurements were taken from distinct samples or whether the same sample was measured repeatedly
☐	☒	The statistical test(s) used AND whether they are one- or two-sided<br>*Only common tests should be described solely by name; describe more complex techniques in the Methods section.*
☐	☒	A description of all covariates tested
☐	☒	A description of any assumptions or corrections, such as tests of normality and adjustment for multiple comparisons
☐	☒	A full description of the statistical parameters including central tendency (e.g. means) or other basic estimates (e.g. regression coefficient) AND variation (e.g. standard deviation) or associated estimates of uncertainty (e.g. confidence intervals)
☒	☐	For null hypothesis testing, the test statistic (e.g. *F*, *t*, *r*) with confidence intervals, effect sizes, degrees of freedom and *P* value noted<br>*Give P values as exact values whenever suitable.*
☒	☐	For Bayesian analysis, information on the choice of priors and Markov chain Monte Carlo settings
☒	☐	For hierarchical and complex designs, identification of the appropriate level for tests and full reporting of outcomes
☒	☐	Estimates of effect sizes (e.g. Cohen's *d*, Pearson's *r*), indicating how they were calculated

Our web collection on statistics for biologists contains articles on many of the points above.

Software and code

Policy information about availability of computer code

Data collection	WES was performed by using formalin-fixed paraffin-embedded (FFPE) sections of pretreatment tumor samples. After pathology assessment, tissue was scraped from the entire section with a fresh scalpel and transferred to a 1.5 mL tube containing 200 µL of 100% ethanol. DNA was isolated using the QIAamp DNA FFPE Tissue Kit (Qiagen, Valencia, CA, USA). Thereafter, tumor DNA was quantitated using the Qubit assay (Invitrogen, Carlsbad, California, USA), and quality was assessed using the QuantideX qPCR DNA QC Assay (Asuragen, Austin, TX, USA). Matched normal DNA was extracted from whole blood collected in a PAXgene Blood DNA Tube (Qiagen) at clinical sites and stored at –20°C or –70°C/80°C until processed in an approved central laboratory identified by the sponsor. The Chemagic STAR DNA Blood Kit (PerkinElmer, Waltham, MA, USA) run on either a Hamilton Chemagic STAR or PerkinElmer Chemagic 360 automated instrument was used to extract DNA in a final volume of 500 µL or 1.0 mL
Data analysis	cDNA levels were assessed using the next-generation sequencing–based GuardantOMNI assay. Four metrics monitoring change in ctDNA levels were evaluated using the ratio of on-treatment cycle 2 (C2) ctDNA levels compared with the pretreatment cycle (C1): Tumor-informed maximum VAF (maxVAF), which used paired tissue and matched normal WES to filter putative somatic variants in ctDNA via confirmation from tissue WES; Tumor-uninformed maxVAF, which did not use paired WES to filter putative somatic variants in ctDNA; Tumor-uninformed mean VAF (meanVAF), which was the average of VAF for somatic mutations restricting attention to those that occurred at C1 (so later variants that might be cleared post-treatment would be included as zeros in the average); and Guardant Health molecular response (MR) score, which can only quantify ctDNA change (ie, cannot be derived at baseline).

For manuscripts utilizing custom algorithms or software that are central to the research but not yet described in published literature, software must be made available to editors and reviewers. We strongly encourage code deposition in a community repository (e.g. GitHub). See the Nature Portfolio guidelines for submitting code & software for further information.

Data

Policy information about availability of data

All manuscripts must include a data availability statement. This statement should provide the following information, where applicable:

- Accession codes, unique identifiers, or web links for publicly available datasets
- A description of any restrictions on data availability
- For clinical datasets or third party data, please ensure that the statement adheres to our policy

Merck Sharp & Dohme LLC, a subsidiary of Merck & Co., Inc., Rahway, NJ, USA (MSD) is committed to providing qualified scientific researchers access to anonymized data and clinical study reports from the company's clinical trials for the purpose of conducting legitimate scientific research. MSD is also obligated to protect the rights and privacy of trial participants and, as such, has a procedure in place for evaluating and fulfilling requests for sharing company clinical trial data with qualified external scientific researchers. The MSD data sharing website (available at: http://engagezone.msd.com/ds_documentation.php) outlines the process and requirements for submitting a data request. Applications will be promptly assessed for completeness and policy compliance. Feasible requests will be reviewed by a committee of MSD subject matter experts to assess the scientific validity of the request and the qualifications of the requestors. In line with data privacy legislation, submitters of approved requests must enter into a standard data-sharing agreement with MSD before data access is granted. Data will be made available for request after product approval in the United States and the European Union or after product development is discontinued. There are circumstances that may prevent MSD from sharing requested data, including country or region-specific regulations. If the request is declined, it will be communicated to the investigator. Access to genetic or exploratory biomarker data requires a detailed, hypothesis-driven statistical analysis plan that is collaboratively developed by the requestor and MSD subject matter experts; after approval of the statistical analysis plan and execution of a data-sharing agreement, MSD will either perform the proposed analyses and share the results with the requestor or will construct biomarker covariates and add them to a file with clinical data that is uploaded to an analysis portal so that the requestor can perform the proposed analyses.

Human research participants

Policy information about studies involving human research participants and Sex and Gender in Research.

Reporting on sex and gender	Extended Data Table 1
Population characteristics	Extended Data Table 1
Recruitment	Details of the trial design and the eligibility criteria have been published (Powles, T., et al. Lancet Oncol. 2021; 22: 931-945). Key eligibility criteria included patients aged ≥18 years with previously untreated locally advanced, unresectable, or metastatic UC; an Eastern Cooperative Oncology Group performance status (ECOG PS) score of 0 to 2; and one or more measurable lesions per Response Evaluation Criteria in Solid Tumors (RECIST) v1.1 by investigator assessment. Sex of participants was determined based on self-report.
Ethics oversight	The study protocol and all amendments were approved by the institutional review board or ethics committee at each participating institution. The study was conducted in accordance with the protocol, its amendments, and the ethical principles originating from the Declaration of Helsinki and Good Clinical Practice guidelines. Written informed consent was provided by all patients before enrollment.

Note that full information on the approval of the study protocol must also be provided in the manuscript.

Field-specific reporting

Please select the one below that is the best fit for your research. If you are not sure, read the appropriate sections before making your selection.

☒ Life sciences ☐ Behavioural & social sciences ☐ Ecological, evolutionary & environmental sciences

For a reference copy of the document with all sections, see nature.com/documents/nr-reporting-summary-flat.pdf

Life sciences study design

All studies must disclose on these points even when the disclosure is negative.

Sample size	260 had evaluable ctDNA at baseline (pembrolizumab, n = 130; chemotherapy, n = 130), and 238 had ctDNA data evaluable at both baseline and C2 (pembrolizumab, n = 115; chemotherapy, n = 123).
Data exclusions	Please see Powles, T., et al. Lancet Oncol. 2021; 22: 931-945 for full information of exclusion criteria from the primary analysis.
Replication	Please see Powles, T., et al. Lancet Oncol. 2021; 22: 931-945 for full information on replication from the primary analysis.
Randomization	Yes
Blinding	Double-Blind

Reporting for specific materials, systems and methods

We require information from authors about some types of materials, experimental systems and methods used in many studies. Here, indicate whether each material, system or method listed is relevant to your study. If you are not sure if a list item applies to your research, read the appropriate section before selecting a response.

Materials & experimental systems

n/a	Involved in the study
☒	☐ Antibodies
☒	☐ Eukaryotic cell lines
☒	☐ Palaeontology and archaeology
☒	☐ Animals and other organisms
☐	☒ Clinical data
☒	☐ Dual use research of concern

Methods

n/a	Involved in the study
☒	☐ ChIP-seq
☒	☐ Flow cytometry
☒	☐ MRI-based neuroimaging

Clinical data

Policy information about clinical studies

All manuscripts should comply with the ICMJE guidelines for publication of clinical research and a completed CONSORT checklist must be included with all submissions.

Clinical trial registration	clinicaltrials.gov (NCT02853305)
Study protocol	Please see Powles, T., et al. Lancet Oncol. 2021; 22: 931-945 for full information on the full study protocol.
Data collection	Please see Powles, T., et al. Lancet Oncol. 2021; 22: 931-945 for full information on patient data collection from the primary analysis.
Outcomes	Primary prespecified objectives for this exploratory ctDNA analysis were to determine whether baseline or on-treatment changes in ctDNA levels, as captured by these metrics, were associated with clinical outcomes (BOR, PFS, and OS). Secondary objectives were to evaluate whether baseline and change-from-baseline ctDNA metrics were independently associated with clinical outcomes in models adjusted for biomarker subgroup, other baseline prognostic factors (baseline tumor burden and ECOG PS), and radiographic response by RECIST v1.1. Please see Powles, T., et al. Lancet Oncol. 2021; 22: 931-945 for full information on primary and safety end points from the full clinical study.
